# Energy barriers at grain boundaries dominate charge carrier transport in an electron-conductive organic semiconductor

**DOI:** 10.1038/s41598-018-33308-y

**Published:** 2018-10-05

**Authors:** I. Vladimirov, M. Kühn, T. Geßner, F. May, R. T. Weitz

**Affiliations:** 1 BASF SE, FET Systems, Carl-Bosch-Straße 38, 67056 Ludwigshafen, Germany; 2 InnovationLab GmbH, Speyerer Str. 4, 69115 Heidelberg, Germany; 3 Physics of Nanosystems, Faculty of Physics, Ludwig-Maximilians University, Amalienstr. 54, 80799 Munich, Germany; 4 Center for Nanoscience (CeNS), Ludwig-Maximilians University Munich, Schellingstr. 4, 80799 Munich, Germany; 5 Nanosystems Initiative Munich (NIM), Schellingstr. 4, 80799 Munich, Germany

## Abstract

Semiconducting organic films that are at the heart of light-emitting diodes, solar cells and transistors frequently contain a large number of morphological defects, most prominently at the interconnects between crystalline regions. These grain boundaries can dominate the overall (opto-)electronic properties of the entire device and their exact morphological and energetic nature is still under current debate. Here, we explore in detail the energetics at the grain boundaries of a novel electron conductive perylene diimide thin film. Via a combination of temperature dependent charge transport measurements and ab-initio simulations at atomistic resolution, we identify that energetic barriers at grain boundaries dominate charge transport in our system. This novel aspect of physics at the grain boundary is distinct from previously identified grain-boundary defects that had been explained by trapping of charges. We furthermore derive molecular design criteria to suppress such energetic barriers at grain boundaries in future, more efficient organic semiconductors.

## Introduction

Progress in understanding charge transport in organic semiconductors has benefited significantly from both experimental and theoretical works. One parameter to assess the quality of charge transport in organic semiconductors is the charge carrier mobility µ of electrons or holes. Significant advancement has been made to increase µ by means of sophisticated synthesis^1–3^, thin film-crystallization^4–7^ and device engineering^8^. This combined effort has enabled reliable mobilities^9^ above 1 cm²/Vs of both electrons^1^ and holes^3^. On the theoretical side, the understanding of parameters that lead to efficient charge transport has also significantly advanced, and different models have evolved to explain mobility data of highly crystalline organic semiconductors (via transient localization^10,11^ or band-like transport^12^). In realistic devices, traps for charges are usually present^13^. One way to classify such traps is by their energetic position with respect to the transport level, namely into valleys (i.e. states of lower energy with respect to the transport level) and barriers (i.e. higher energy states). Additionally, both valleys and barriers can either be shallow i.e. they are within few *k*_*b*_*T* of the transport levels (e.g. shallow valleys are partially depopulated at room temperature) or they can be deep i.e. at energies significantly lower/higher than *k*_*b*_*T*. Charges do not typically escape deep valleys at room temperature and once trapped, such carriers lead to potential barriers for other carriers of the same type due to their mutual Coulomb repulsion^14,15^.

The relative impact of traps on the transistor characteristics is still under current debate and typically most experimental works focus on the role of the valleys. While for example Podzorov *et al*.^16^ and Li *et al*.^17,18^ point out that deep filled valleys mainly impact the threshold voltage (*V*_*th*_) but not µ, Xie *et al*.^19^ and Zhang *et al*.^20^ find that they impact µ. The situation is similar with shallow traps, where trapped majority carriers have been attributed to only impact *V*_*th*_ but not µ^21^, or to only impact µ^17,18^. The situation is further complicated by the dependence of the height of the filled valleys on the density of free charge carriers. The reason is that the energy barriers caused by such filled wells can be screened by ionized dopants or counter charges at the gate dielectric thus reducing the effective trap height^14,15,22,23^. To complicate things even further, traps can have different origin and can for example stem from grain boundaries in the organic semiconductor^14,15,23–29^ or be located at the interface of the semiconductor to the dielectric^30,31^.

While general strategies are known to passivate traps in the dielectric e.g. via self-assembled monolayers^32^, grain boundaries in thin films of small molecules deposited by vapor or solution processing can often times not be avoided, especially when thinking of large-scale processing (even though there are promising approaches how to avoid them at the laboratory scale^4–7^). It is therefore important to understand the implications of grain boundaries in small molecular thin films on charge transport. In realistic thin film transistors, grain boundaries can dominate charge transport, since they can act as traps for charges thus reducing the charge carrier mobility^33,34^, lead to increased bias stress^35,36^ or lower long-term electrical stability^37,38^. Additionally, the impact of grain boundaries seems to be especially strong if the semiconductor is only one monolayer thin^29^. Besides this general understanding of the overall detrimental impact of grain boundaries on charge transport, a consistent microscopic picture of grain boundaries is just emerging. For example, there is no clear picture of their impact on the energetic landscape which is reflected by the density of states (DOS) of semiconducting films. More specifically, while experimental works have identified that large angles between adjacent crystals lead to larger trap energies for charge carriers^39–41^ or excitons^42^, no such clear angular dependence was found in theoretical studies^26^. To increase performance of organic thin film transistors, knowledge of design criteria to limit the impact of grain boundaries e.g. via deterministic molecular design would be very advantageous.

In general, it is known that, depending on microscopic details, grain boundaries can lead either to a deep valley^36,43^, a shallow valley^40,44^ or to a shallow or high^28^ energy barrier for charge carriers^18,25–27^. Here, as discussed above, the deep valleys once filled with the majority carriers turn into barriers^45^ with the barrier height depending on the density of free carriers^14,15,22,46,47^. Also, traps at grain boundaries are known to impact *V*_*th*_ and µ. However, in contrast to the traps at the semiconductor/dielectric interface, grain boundaries typically percolate through the entire transistor channel, meaning that charge carriers typically have to cross at least one grain boundary on their way through the channel. This is different than in the case for charge traps that do not stem from grain boundaries and thus can be assumed to be more or less randomly distributed through the film, so that charges can always find a way around such traps. In practice, this means that if the grain boundary density is high, all free charge can be trapped at the grain boundary leading to a depletion of the grain itself^15,46^. Finally, in the literature it is typically assumed, that the main mechanism that limits µ upon transport through a grain boundary are energy barriers due to trapped majority carriers^14,15,45,46,48^. Again, up to now no attention has been paid to the role of energy barriers at grain boundaries that do not originate from filled valleys, even though one could imagine them to have a dramatic impact on charge transport in the case that they are located at a grain boundary that percolates across the entire channel.

One important parameter to compare theoretical to experimental works is knowledge of the density of states (DOS)^13^ which reflects the energy landscape of semiconducting films. Both, theory and experiment show, that depending on microscopic parameters the DOS follows either a Gaussian or exponential distribution, or both^20^. While a Gaussian DOS is frequently assigned to disorder due to fluctuating van-der-Waals contacts within a single crystalline region^49^, an exponential DOS is generally attributed to disorder at connection sites between crystalline regions i.e. grain boundaries^43^ or sometimes also to thermal fluctuations of the small molecules^50^. One can therefore expect to gain insight into the energetics at grain boundaries by measuring the DOS for various grain boundary densities.

Here, we present a detailed experimental and theoretical investigation to add more systematics to the relation between the density of grain boundaries and transport parameters. To this end, we compare in detail the electrical characteristics of a high-performance electron conductive organic small-molecule semiconductor to theoretical models of charge transport in polycrystalline monolayers of this semiconductor. This allows us also to shed light on the currently unexplored influence of the energy barriers at grain boundaries on charge transport.

**Figure 1:**
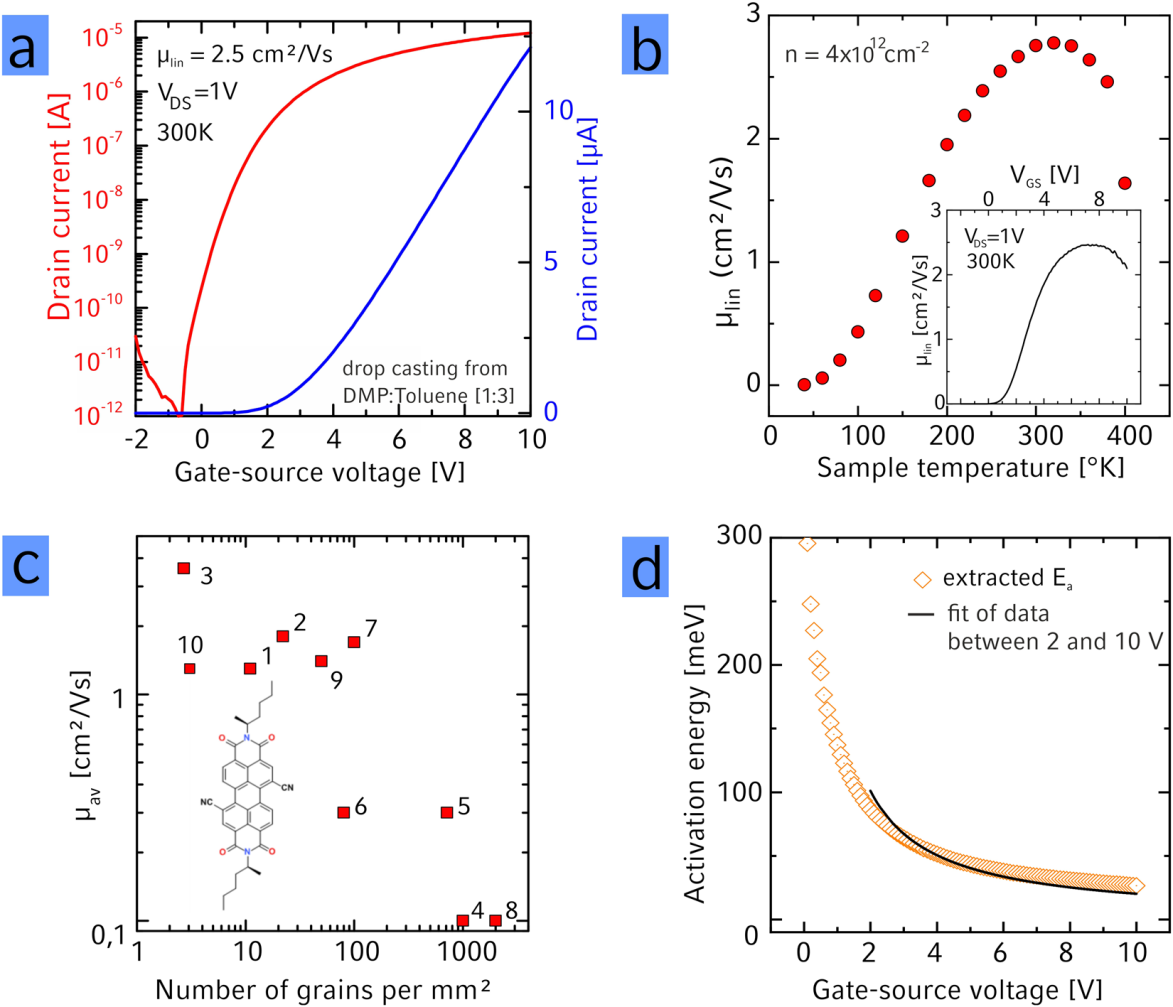
Transfer (**a**) characteristics and (**b**) temperature dependence of the linear mobility in a 3 nm thin organic semiconducting thin film (inset: gate dependence of the linear mobility of the same transistor). (**c**) Average extracted linear charge carrier mobility as function of the number of grains. The solvents used for deposition of the semiconductor are: 1: DEP, 2: DAP, 3: DMP:Tol (1:3), 4: DMP:AmAc (1:3), 5: DAP:AcAc (1:3), 6: DAP:AmAc (1:3), 7: DMP:AcAc (1:9), 8: DMP:AcAc (1:49) 9: DMP:AcAc (1:3), 10: DMP (inset: structure of PDI1MPCN2). (**d**) Activation energy *E*_*a*_ as function of V_GS_ for the data shown in (**b**) obtained by fitting an Arrhenius law to the temperature dependence of the linear charge carrier mobility.

The experimental part of our study is performed using thin films of the high-performance semiconductor PDI1MPCN2 (N,N′-di((S)-1-methylpentyl)-1,7(6)-dicyano-perylene-3,4:9,10-bis(dicarboximide)) (Fig. 1c (inset)), which we recently showed to possess linear charge carrier mobilities µ as high as 4.3 cm²/Vs in only 3 nm thin, highly crystalline thin films^6^. We attribute the overall large mobility to the high crystalline order as well as to the absence of significant thermal fluctuations (i.g. dynamic disorder). This is because the latter turn out to be energetically more unfavorable for the twisted cores than for the planar cores due to the intercalation in packing that prevents sliding (see supplementary information, Figure S6)^11,51,52^. A typical transfer curve measured in vacuum is shown in Fig. 1a. To evaluate the nature of charge transport in the highly crystalline films, we have also performed temperature dependent measurements of µ (Fig. 1b, temperature dependent transfer curves are shown in Figure S1a in the SI). Between room temperature and 40 K the mobility decreases exponentially, which allows us to extract an activation energy *E*_*a*_ as detailed below. In short, in the highest crystalline films we find activation energies as low as 20 meV, underlining the high crystal quality of these films. At temperatures above ~300 K the charge carrier mobility decreases as previously observed in single crystalline devices for example of the perflourinated perylene diimide PDIF-CN_2_. There, the decrease in mobility at elevated temperatures was attributed to intrinsic charge transport in the semiconductor^53^, potentially caused by dynamic disorder^11^. For the remainder of this work we focus on the temperature range below RT.

In our previous work we systematically identified that using solvents of high surface tension and high viscosity allows for crystallization of the semiconductor at the liquid-air interface, and leads to highly crystalline films with domains sizes as large as 100 µm² ^6^. In the present work, we have used this method to realize films with higher grain boundary density via the addition of solvents with lower viscosity and surface tension and studied their electrical performance in detail. We were able to vary the grain boundary density across three orders of magnitude (from ca. 1 to 1000 grains/mm² as identified by polarized optical microscopy, for detailed images are displayed in the Supplementary Information of ref.^6^). For the deposition of our thin films from various solvents, we only have changed the solvent composition but left other transistor fabrication parameters the same. This makes us confident, that the observed changes in electrical characteristics with varying crystal size are only due to this change in morphology and not due to other changes. Along with the decrease in grain size, µ was found to decrease from maximum values of 4.3 cm²/Vs (using a mixture of toluene:dimethylphthalate (1:3 weight ratio, details in the experimental section)) in the best quality films all the way down to 0.1 cm²/Vs in films containing a significant amount of grain boundaries (Fig. 1c). The strong dependence of µ on the density of grain boundaries is expected and has been experimentally verified multiple times, This is typically explained by the presence of shallow or deep valleys trapping charge carriers at the grain boundary^40,44,46–48^.

**Figure 2:**
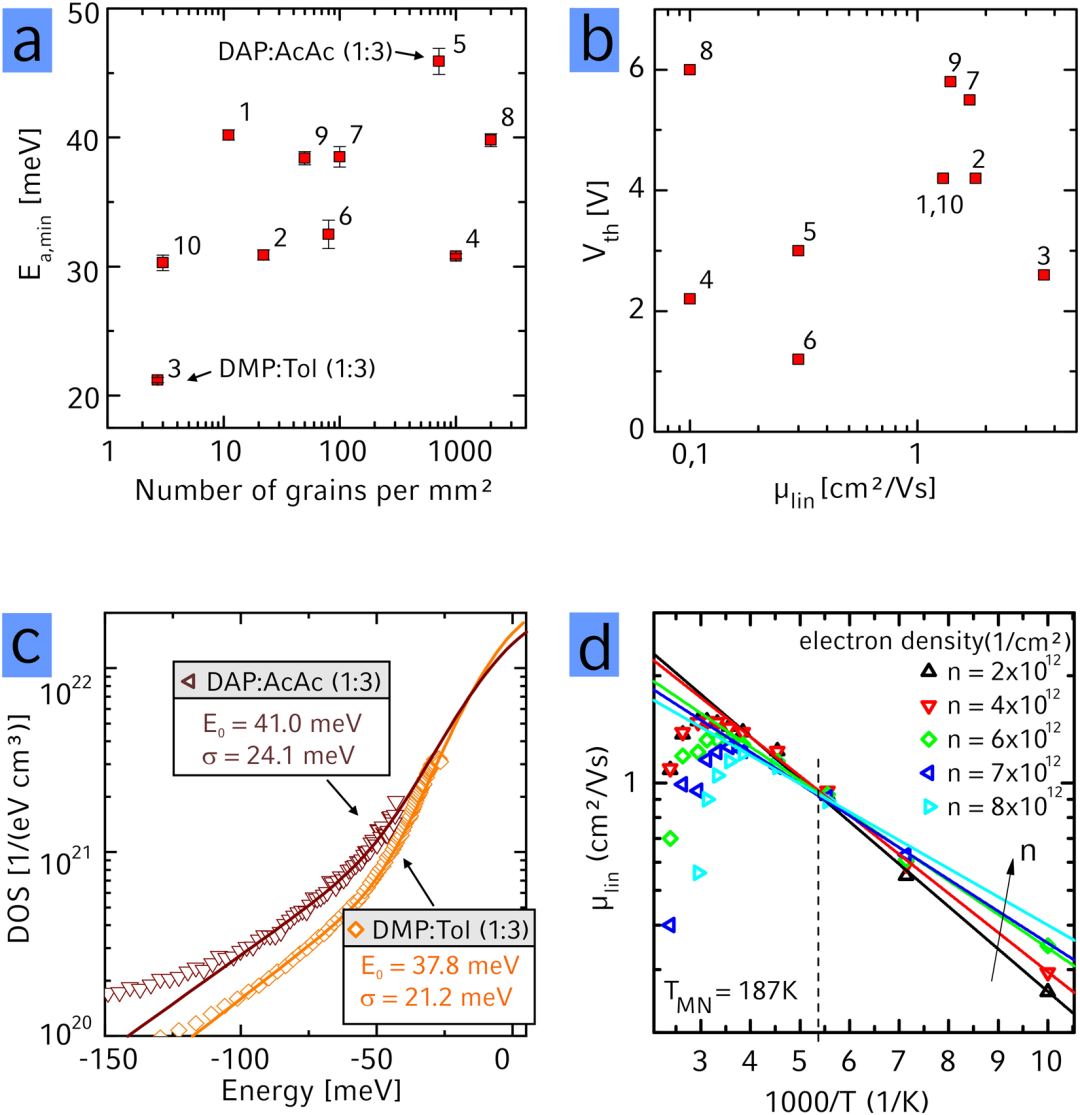
(**a**) Minimal activation energy as function of number of grains. No clear increase of the activation energy with increasing grain density can be seen. (**b**) Relation between threshold voltage and linear mobility for thin films deposited from various solvent mixtures No clear relation between the threshold voltage and the mobility can be observed. The solvents used for deposition of the semiconductor are: 1: DEP, 2: DAP, 3: DMP: Tol (1:3), 4: DMP:AmAc (1:3), 5: DAP:AcAc (1:3), 6: DAP:AmAc (1:3), 7: DMP:AcAc (1:9), 8: DMP:AcAc (1:49) 9: DMP:AcAc (1:3), 10: DMP. (**c**) Density of states (DOS) obtained from *E*_*a*_*(V*_*GS*_) curves based on highly crystalline films of PDI1MPCN2 obtained from DMP:Toluene (orange) and less crystalline films from DAP:AcAc (brown). Solid lines are a fit to the experimental data using a combination of Gaussian and exponential DOS as described in the text. Fit values are for the Gaussian part σ_DMP:Tol_ = 21.2 meV, N_0,DMP:Tol_ = 1.8 × 10²² 1/(eVcm³), σ_DAP:AcAc_ = 24.1 meV, N_DAP:AcAc_ = 1.47 × 10^22^ 1/(eVcm³) and for the exponential part: E_0,DMP:Tol_ = 37.8 meV, N_0,DMP:TOL_ = 3.51 × 10^21^ 1/(eVcm³), E_0,DAP:AcAc_ = 41.0 meV, N_0,DAP:AcAc_ = 5.1 × 10^21^ 1/(eVcm³). (**d**) Plot of linear mobility vs 1/T for various carrier densities. At low temperatures, the mobility follows an activated behavior and the linear extrapolations of this activated behavior for different charge carrier densities cross at a particular temperature as discussed in the text.

In order to understand the impact of grain boundaries on charge transport in more depth, we have extracted the activation energy *E*_*a*_ from the temperature dependence of the linear charge carrier mobility *µ*_*lin*_ (Fig. 1b) as function of the gate-source voltage *V*_*GS*_ for transistors composed of semiconductor films of various crystallinity (see Figure S1 for µ(T) data of films of lower crystallinity). First, we would like to note, that the charge carrier mobility does not strongly depend on the gate voltage at room temperature, for exemplary graphs see the inset to Figs 1b and S1 – an indication that our mobility measurements are not contact limited^9,54^. For our analysis of our temperature dependent charge transport data, we have first used the multiple trap and release model^46^ – see for details on the alternative variable-range hopping model in e.g. ref.^55^. According to the multiple trap and release model^46^, the mobility should vary with temperature as *µ* = *µ*_0_ *exp(−E*_*a*_*/k*_*b*_*T)* with *µ*_0_ the band mobility, *k*_*b*_ Boltzmann’s constant and *T* the temperature. The *E*_*a*_ vs *V*_*GS*_ trace for the same transistor shown in Fig. 1a and b is displayed in Fig. 1d and the minimal value of *E*_*a*_ for various crystallinities investigated is shown in Fig. 2a. The *E*_*a*_ vs *V*_*GS*_ traces for all transistors investigated is shown in the SI, Figure S2. For a larger density of grain boundaries also the minimal *E*_*a*_ slightly increases in principle, consistent with the general understanding that traps at grain boundaries can dominate *E*_*a*_^15,46^. The general relation between *E*_*a*_ and the grain boundary density leads us to conclude that the *E*_*a*_ – at least in the less ordered films - is dominated by the grain boundaries and not by other sources of disorder like interaction with the dielectric e.g. in the form of Fröhlich polarons as could be expected when dielectrics with a high dielectric constant are used, such as *Al*_2_*O*_3_ utilized in our work^56^. Additionally, we have also manufactured transistors using no SAM as between the Al_2_O_3_ and the organic semiconductor and transistors using SiO_2_ as dielectric and have not found a systematic dependence between the dielectric constant and the charge carrier mobility. We therefore believe it is safe to assume that the transport properties are dominated by the grain boundaries and we can use these temperature-dependent charge transport measurements to learn about their nature.

While the general trend that *E*_*a*,*min*_ varies with the density of grain boundaries seems consistent with literature, the large mobility suppression with increasing density of grain boundaries cannot be explained by the *E*_*a*,*min*_ obtained from the samples of different grain boundary density. More specifically, the variation of µ from 0.1 to 4.3 cm²/Vs is too large to be caused by a variation of *E*_*a*,*min*_ between 20 and 50 meV. This implies that *E*_*a*,*min*_ is not a good indicator to describe the impact of grain boundaries on µ in our system. Another way to test whether the model in which trapped charges at the grain boundary can describe our data, is to check the dependence of *E*_*a*_ on the gate voltage. As described above, the effective barrier height at the grain boundaries *E*_*b*_ (which in this model corresponds to *E*_*a*_) depends on the density of grain boundaries and the charge carrier density induced by *V*_*GS*_ or by unintentional dopants^14,15^. This has two important consequences. First, the presence of unintentional dopants should then (besides decreasing *V*_*th*_ in electron conductive materials with increased density of dopants) also lead to an increase of mobility. This is however not seen in our experiments, where *V*_*th*_ does not seem to correlate with *µ* (Fig. 2b)^21^. Second, the actual density of traps can be extracted from the *E*_*a*_ vs. *V*_*GS*_ trace, since at high charge carrier density *E*_*a*_ depends inversely proportional on *V*_*GS*_ (${E}_{a}=\frac{{e}^{2}{n}_{t}^{2}{d}_{m}}{8{\varepsilon }_{0}{\varepsilon }_{r}C{V}_{GS}}$, with *e* the electrons charge, *n*_*t*_ the density of trap states at the grain boundary, *d*_*m*_ the thickness of the charge sheet (here 1.7 nm (height of one monolayer)), *ε*_0_ the vacuum permittivity, *ε*_*r*_ = 3 the permittivity of the semiconductor and *C* the capacitance of the gate dielectric (here 190 nF/cm²))^15^. As shown in the SI (Figures S2 and S3) we have extracted the trap densities *n*_*t*_ and obtained values between 4 and 6 × 10^12^ 1/cm². These extracted trap densities are very high given that this would correspond to about half of the charge carrier density that can be accumulated with the gate electrode to be trapped. Furthermore, the mobility and also the grain boundary density extracted from optical images do not consistently scale with *n*_*t*_ e.g. the trap density varies only by a factor of two while the number of grains per mm² varies across several orders of magnitude (see SI Figure S3). Since furthermore also *V*_*th*_ does not correlate with µ, we conclude that the model of potential barriers formed by trapped charges at grain boundaries cannot sufficiently explain our data which motivates search for alternative explanations.

A further approach to understand the relation between electrical performance and trap states is via the density of states (DOS). As detailed in the SI (section 1), the gate dependence of *E*_*a*_ can be used to calculate the DOS (Fig. 2c)^57^. Our extracted DOS can be approximated by a combination of a Gaussian small negative energies and an exponential at larger negative energies (Fig. 2c), as also has been found in previous works^20,58^. In general, the Gaussian part of the DOS is assigned to stem from randomly varying intermolecular distortions of the molecules within a grain^49^. The exponential part at comparably lower energies is associated with long-range Coulomb potential fluctuations, thermal motion of small molecules^50^ or disorder at the grain boundaries^43^. A check to the validity of using a Gaussian at high energies to approximate the DOS can be made by testing for a point where the slopes of the linearly extrapolated inverse temperature dependences of µ meet for different charge carrier densities. This isokinetic point is called the Meyer-Neldel temperature and is in our case with *T*_*MN*_ = *187* *K* (Fig. 2d) exceptionally low compared to previous works, indicative of the high crystal quality obtained in our thin films. Also, to the best of our knowledge our work is the first one showing really a crossing point of the experimental data and not only of the extrapolated ln(µ) vs 1/T plots, again indicative of the high sample quality. The observation of this isokinetic point is an indication for the movement of charge carriers in a filled DOS broadened by Gaussian disorder^59^ thus underlining the validity of choosing the Gaussian DOS to approximate our data.

While the Gaussian part of the DOS is typically interpreted to stem from disorder within highly crystalline regions, the exponential part is attributed to be caused by disordered regions at the grain boundary. With our solution-based approach of thin film deposition, we have the possibility to tune the grain boundary density and can test if this density is related to the exponential part of our fits. A list of a wider selection of solvents together with the obtained fit values for the DOS is summarized in Table 1 – the respective temperature dependent *µ*, *E*_*a*_ and obtained DOS data is shown in the SI (Figures S2 and S4). It is noticeable that the fit values are low compared to previous works^19^. We find 35 meV < *E*_0_ < 55 meV and 18 meV < *σ* < 25 meV. Such low values for *E*_0_ and *σ* are consistent with the overall high mobility measured in our films. *E*_0_ is found to correlate well with the threshold voltage *V*_*th*_ consistent with previous reports that deep trapping at grain boundaries impacts on *V*_*th*_^16^. We however did not find a correlation between *E*_0_ or *σ* with neither the density of grain boundaries nor the charge carrier mobility (see SI Figure S4).

**Table 1:** Selection of electric properties of different solvents and solvent mixtures. DMP = dimethyl phthalate; DAP = diallyl phthalate; AcAc = acetylacetone; AmAc = amylacetate.

Solvent [mixing ratio]	Grains per mm²	µ_lin_^max^	µ_lin_^ave^	V_th_^ave^	E_a min_	σ	E_0_
		[cm^2^/Vs]		[V]	[meV]		
DMP:Toluene [1:3]	2.7	4.42	3.6 ± 0.6	2.6 ± 0.1	21.2 ± 0.2	21.2	37.8
DEP	11	1.44	1.0 ± 0.2	4.2 ± 0.2	40.2 ± 0.4	18.3	41
DAP	22	2.74	1.8 ± 0.5	4.2 ± 0.3	30.9 ± 0.4	22.0	50.9
DAP:AmAc [1:3]	80	1.63	0.3 ± 0.3	1.2 ± 0.4	32.5 ± 1.1	19.9	36.7
DMP:AcAc [1:9]	100	2.50	1.7 ± 0.4	5.5 ± 0.4	38.5 ± 0.8	24.4	51.3
DAP:AcAc [1:3]	715	0.31	0.3 ± 0.0	3.0 ± 0.7	45.9 ± 1.0	24.1	41.0
DMP:AmAc [1:3]	1000	0.14	0.1 ± 0.0	2.2 ± 0.4	30.8 ± 0.2	20.6	39.3
DMP:AcAc [1:49]	2000	0.85	0.1 ± 0.1	6.0 ± 0.9	39.8 ± 0.5	21.9	48.3

It is evident that both the direct evaluation of the density dependence of *E*_*a*_ as well as the evaluation of the DOS cannot explain the relation between crystallinity and µ. Both interpretation approaches have in common, that they use data extracted from the temperature dependence of µ. Possibly this model does not capture the entire physical picture. For example, as mentioned above not only valleys or filled valleys lead to scattering of charges, but also barriers in the energy landscape. Possibly investigating them can shed light on our data with greater accuracy.

**Figure 3:**
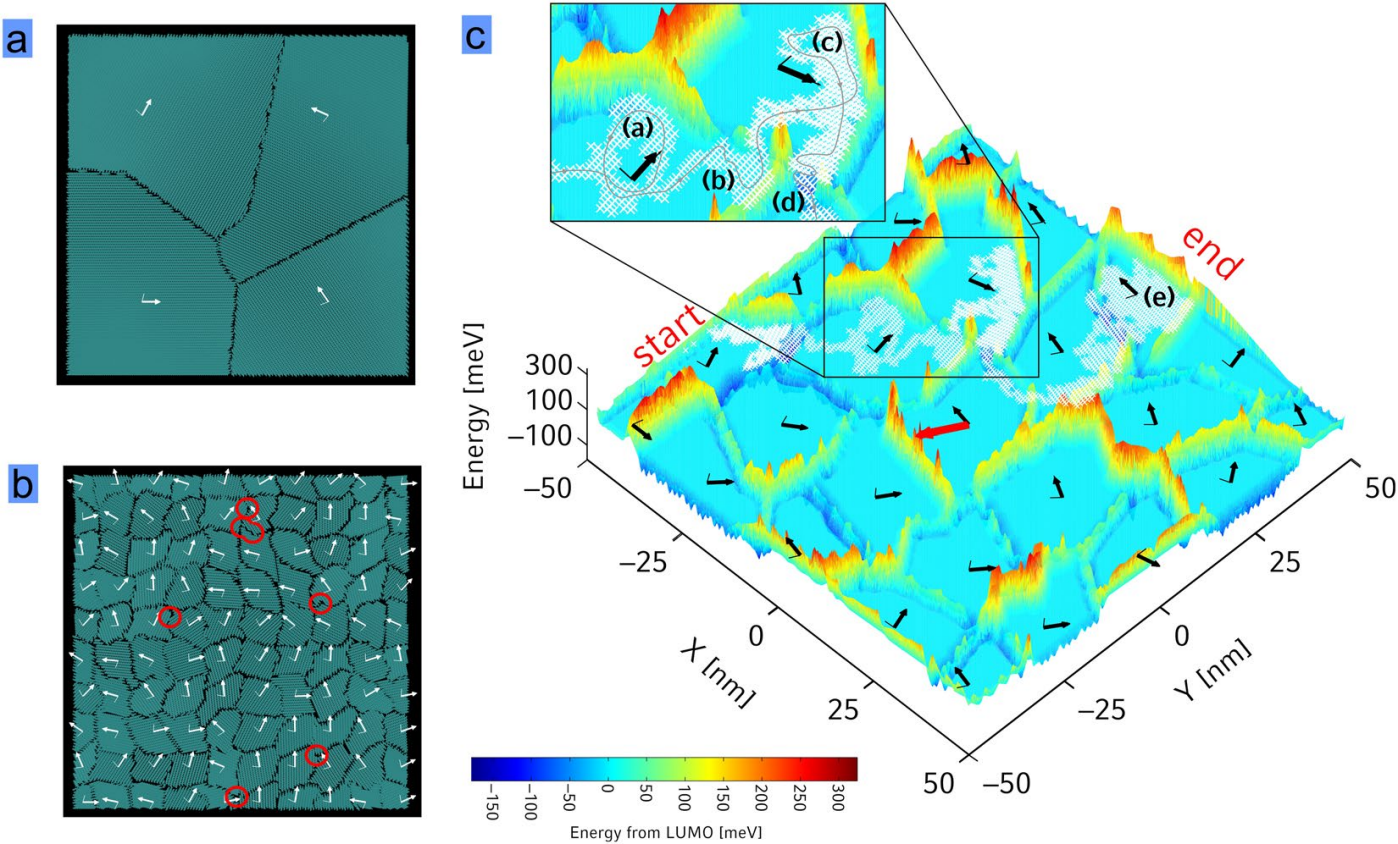
(**a** and **b**) Simulated morphology of a polycrystalline monolayer PDI1MPCN2 film of 2 × 2 and 10 × 10 crystal grains on and area of (100 nm)^2^, with white arrows indicating the crystallographic a-direction (see also supplementary Movie S1). Deepest traps occur close to strongly curved grain boundaries occurring more often for smaller grains (red circles in **b** and **c**) Energetic landscape for localized electrons in a PDI1MPCN2 monolayer consisting of 5 × 5 crystal grains. The colorscale indicates energies relative to the value obtained in the center of the grains. Crystallographic a - and a + b-directions are displayed as bold and thin black arrows, respectively. One exemplary trajectory of a single electron for a field applied along the red arrow is indicated by marking all visited molecules white from start to end (see text for explanation of the part of the trajectory shown in the inset, and supplementary Movie S2 for an animation).

To this end we have performed simulations of monolayers of PDI1MPCN2 with various crystal grain densities in the film and evaluated the DOS and the charge carrier mobility in these films. The details of how the polycrystalline monolayer morphology has been generated is presented in the methods section and the SI. The result of two such morphologies can be seen in Fig. 3a and b.

To evaluate the energetic landscape for electron transport, we have started from the Coulomb interaction between each molecule in the anionic state with the surrounding neutral molecules based on atomistic partial charges obtained by density-functional theory in the gas phase and a relative dielectric constant of *ε* = 3. In detail, we used microscopically computed parameters like intra- and inter-molecular reorganization energies, electronic couplings and site energies all calculated ab-initio based on our monolayer morphologies (details are given in the SI section 2). An example of a resulting energy landscape is given in Fig. 3c setting the electron affinity (LUMO) of molecules in the center of the grains to *E* = 0 (more data is shown in the SI, section 2.3, an energy landscape for the HOMO in section 2.8 in the SI).

**Figure 4:**
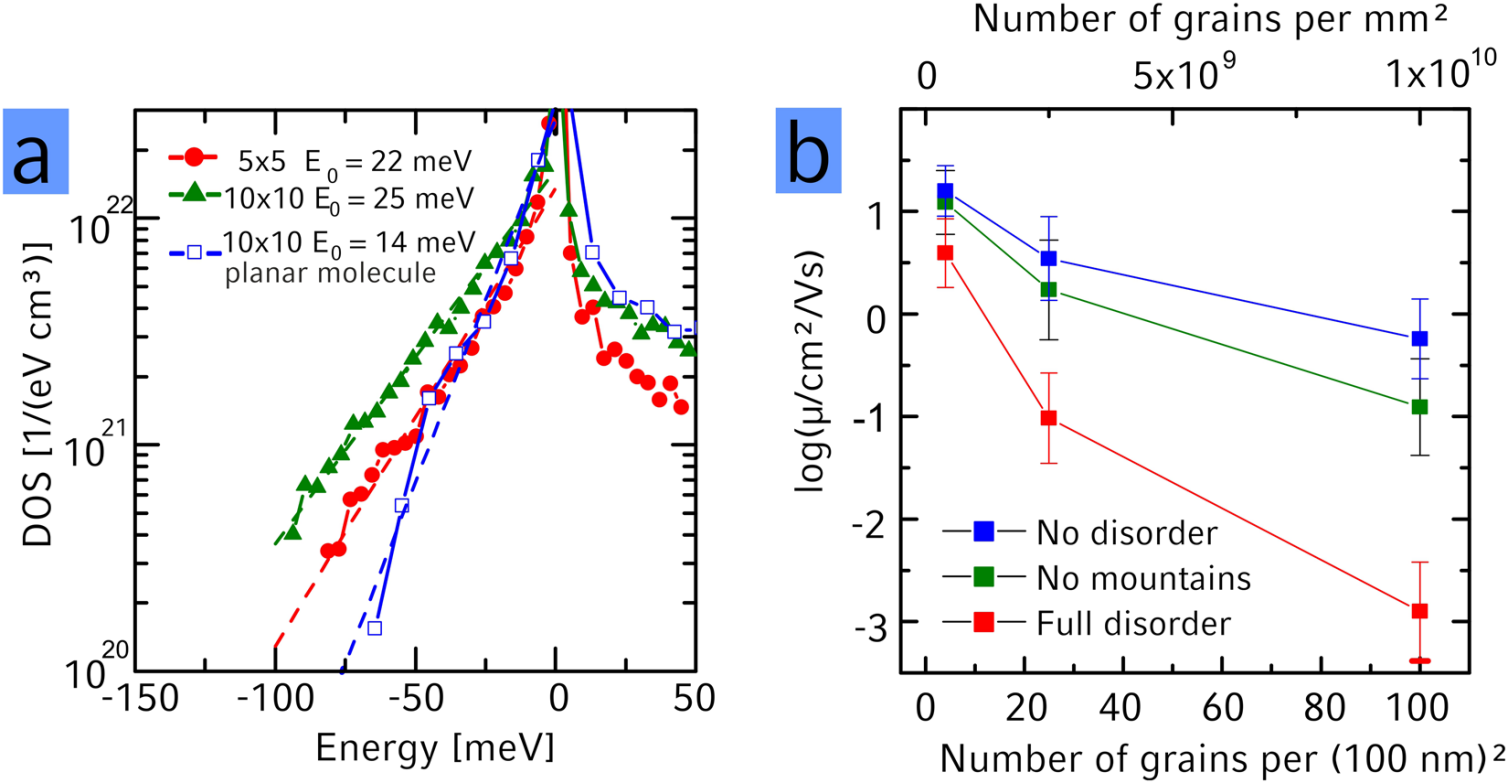
(**a**) Simulated DOS for monolayers of 5 × 5 (red) and 10 × 10 (green) grains on 100 nm^2^ based on Coulomb interaction for localized charges with surrounding neutral molecules. Both curves are asymmetric and show exponentially distributed traps with increasing E_0_ (slower decay) for decreasing degree of crystallinity. (**b**) Simulated average mobility as a function of the degree of crystallinity for full energetic disorder (red), no energetic disorder (blue) and energy landscapes where all activation barriers have been planarized by setting all energies of E > 0 to E = 0 (green).

From the energy landscape, we have calculated the explicit density of states (DOS, Fig. 4a). Since we assumed a perfect crystal within the grains and only derivation from the crystallinity at the grain boundaries, the energy landscape within the grains is flat and for the DOS we focus only on the part at the grain boundaries. We find a non-symmetric DOS with a similar amount of states *E* < 0 (valleys) and *E* > 0 (barriers). Energies fall off faster for valleys compared to barriers. Additionally we find that the variance of the DOS increases with increasing grain boundary density, indicating that charges move through a “rougher” energy landscape. For the valleys our ab-initio results indicate an exponential decay g(*E*) = N*exp(*E*/*E*_0_) as also observed in experiment with increasing *E*_0_ (slower decay) for decreased degree of crystallinity. We attribute this trend to a higher degree of irregularity in the grain edges which can be seen in Fig. 3b: the deepest traps occur close to strongly curved grain boundaries (red circles). We have compared the calculated slopes of the exponential DOS with the slopes of the experimentally determined DOS in the SI (section 2.5) and find reasonable agreement despite the mentioned uncertainties in the validity of the model used for interpretation of the temperature dependent measurements. Additionally, we have compared the experimentally found density located in part of the DOS to the simulations and again find reasonable agreement (SI, section 2.5).

Our ab-initio simulations also reveal that the origin for the energetic disorder created at the grain boundaries is the electrical dipole moment of PDI1MPCN2 (*d* = 1.7 Debye) arising from the cyano groups of the twisted core (for details see SI section 2.6). In case of a planarized core with vanishing dipole moment the DOS decays much faster as shown in Fig. 4a. Synthesis of molecules with a smaller dipole moment should therefore suppress the traps at the grain boundaries, which then, in turn, would of course make the molecular structure more prone to dynamic disorder (see above). Furthermore, we find the trap states to occur most often at grain boundaries where the respective *a*-directions of the grains form an angle close to 180°, a feature that is also related to the asymmetry of the twisted core (see SI section 2.6 for more information about the DOS). This observation is in agreement with previous experimental results showing that higher angle grain boundaries lead to deeper traps^40,41^. However, surprisingly there is no such angular dependence for energy barriers (see SI Figure S16).

Having determined the lateral energy landscape has also allowed us to estimate the mobility of charge carriers in the polycrystalline thin film. The different regimes of charge transport in high-mobility organic semiconductors can range from band-like transport (completely delocalized charges) to hopping (localized charges)^11^. From our experimental observations above (e.g. µ scales with the density of grain boundaries) we deduce that the factor dominating charge transport in our system originates from the grain boundaries, where the carriers localize due to weak electronic overlap and strong energetic disorder. Therefore, we employ a Jortner hopping rate based on microscopically computed parameters (details are given in the SI section 3).

Since we have additionally determined the lateral energy landscape, it is now also possible to assess the relative impact of energy valleys and activation barriers on the overall charge carrier mobility. This was achieved by visualizing the charge carrier trajectory and estimating the mobility through Kinetic Monte Carlo (KMC) simulations based on Jortner hopping rates for single electrons where all parameters are calculated from ab-initio (see SI section 3.2).

A typical electron trajectory is shown in a movie (Supplementary Movie S2) and summarized in Fig. 3c for an applied electric field *F* = 10^7^ V/m (red arrow). All visited molecules are marked white. In the inset, one can see that an electron travelling along the *a*-direction is reflected by an energetic barrier (a), breaks through a grain boundary at a low-energy site (b), is again reflected at a barrier (c), is trapped at a grain boundary (d) and finally is trapped by three energy barriers (e). Mobilities with corresponding errorbars for different degrees of crystallinity in Fig. 4b are obtained from averaging over two different realizations of the morphologies, 18 electrical field directions within the (*a*,*b*)-plane and 50 different electron injection points. We have addressed the influence of the energetic disorder at the grain boundaries on the transport properties by comparing three disorder scenarios in Fig. 4b. First of all one can see, that the mobility reduction with decreased crystallinity is only weak when energetic disorder is turned off completely by setting all *E* = 0 (blue) leaving only electronic couplings as a source of disorder. Since those couplings are of equal magnitude along the *a*- and *a* + *b*-direction the carrier can easily travel also perpendicular to a grain boundary such that the disorder in electronic couplings at the grain boundary is only weakly affecting the mobility (for a comparison to the one-dimensional transport scenario see SI section 3.2.2).

Besides the case of zero energetic disorder (blue) and the original full energetic disorder (red) we planarized only the activation barriers by setting all energies *E* > 0 to *E* = 0 in the simulated DOS (green). Surprisingly, introducing only the valleys (but not the barriers) reduces the mobility only slightly (blue to green), while including also the barriers has a much stronger effect (green to red). This shows that the high-energy states introduced by the grain boundaries play an important role in charge transport by blocking pathways in the monolayer (see also inset of Fig. 3c).

The observation that mostly the high-energy barriers limit the charge carrier mobility is an important point that justifies the applicability of the presented charge transport calculations: While our calculations have been obtained with single carrier occupancy, our experiments that are performed at finite charge carrier density. However, as has been argued previously^14,23^, traps at grain boundaries are typically populated with charges in realistic devices. Such a filled trap in turn leads to the repulsion of other charges and consequently to a potential well. At finite charge carrier density however, charges in the gate electrode will screen this potential well mostly, leading to a strong suppression of the potential well formed at the filled trap. Energy barriers on the contrary, are however not filled (i.e. remain charge neutral) and are consequently not screened. This means, that the values obtained in our modeling where we did not consider finite density effects, are also to be expected to hold in the finite density regime of realistic devices. The role of valleys on the other hand seems to be limited – both in the single particle modeling results obtained here and in experiment (due to screening as described above).

From our extensive theoretical and experimental study of charge transport in thin films of PDI1MPCN2 we can derive several conclusions relevant for future experiments. We have used the temperature dependence of the linear charge carrier mobility for thin films of various crystallinity to derive the charge carrier density dependent activation energy and from it the density of states. The charge carrier density dependent activation energy was found to be inconsistent with the model of trapped charges causing potential wells for charges contributing to the mobility. We could describe the density of states by the combination of a Gaussian part at energies close to the transport level and an exponential at comparably lower energies. Neither the charge carrier mobility nor the density of grain boundaries was found to correlate with the features of the DOS. Also the direct evaluation of the activation energy could not explain our data. Both of these approaches use the model of trapped charge carriers to explain the temperature dependences of µ. Possibly, the model of charge carrier trapping does not capture the entire physics. We therefore used a combination of density-functional theory and kinetic Monte-Carlo simulations of charge transport in polycrystalline monolayers of the PDI1MPCN2 to understand our charge transport results. We found, that due to the misalignment of molecules at grain boundaries energy barriers are created that decisively determine the charge carrier mobility. On the other hand we identified, that valleys in the energy landscape only play a minor role. This result is significant, since previously only valleys or energy barriers created by filled traps have been considered to hinder charge transport. Furthermore, the lateral energy landscape turned out to be more decisive than the transfer integral between the molecules. Finally, we have also identified in the dipole moment due to the twist angle of the PDI1MPCN2 core a molecular factor that has significant impact on the disorder at the grain boundaries. Since the twisted core was found to suppress dynamic disorder and therefore lead to high mobilities within highly crystalline regions, it will be an interesting future challenge to identify molecules that have small dipole moments yet cores that suppress thermal disorder. Furthermore, we believe that our results are also of interest for the improvement of organic solar cells and organic light-emitting diodes, since in these devices typically a significant number of grain boundaries are present.

## Materials and Methods

### Transistor preparation and electrical measurement

Transistors were prepared as previously described^6^ on degenerately doped Si wafers as substrate and gate electrode with a 30 nm Al_2_O_3_ gate oxide grown by atomic layer deposition modified with a patterned SAM. We used thermally evaporated top gold source and drain contacts patterned through a shadow mask thus defining the channel with a length of 50 µm and a width of 200 µm. Charge transport measurements were performed in a Lakeshore variable temperature probe station in high vacuum. The dielectric constant of the bare Al_2_O_3_ films and the SAM-treated substrates were determined to be 7.4 and 6.6, respectively.

### Simulation of grain boundary morphology

On an area of 100 nm^2^ we have created different monolayers consisting of NxN crystal grains (N = 2, 5, 10, 15) with periodic boundary conditions in the transport plane (a,b) by choosing equidistant seeding points with randomly chosen a-directions (see Supplementary Movie S1). Crystals are grown in a spherical fashion in accordance with the experimental unit cell, where the only unknown is the molecular orientation. We have assumed an orientation which complies with isotropic transport and van der Waals energies (see SI, where we also compare to an orientation leading to non-isotropic transport). Furthermore, isotropic and uniform growth speed was assumed for the crystal grains. Atoms within molecules from neighboring grains were allowed to come as close as 2 Å. Relaxation of side chains at the grain boundaries was taken into account by shortening them to isopropyl in order to capture sterical effects.

### Fit of the density of states (DOS)

To approximate the DOS we assumed a sum of an exponential and a Gaussian:

2 $${\rm{DOS}}={N}_{{\rm{0}}}{e}^{(E+{\rm{\Delta }})/{E}_{0}}+N{e}^{-{(E+{\rm{\Delta }})}^{2}/2{\sigma }^{2}}$$

First, the DOS at large negative energies (deep traps, between $E=-100$ meV and $E=-70$ meV for the raw data) is regarded, which can be described by an exponential. A linear fit of the logarithmized DOS then yields the parameters ${N}_{\exp }$ and ${E}_{0}$. Second, the fitted exponential term (see equation above) is subtracted from the measured DOS for all energies. The resulting DOS at small negative energies (shallow traps) can then be attributed to a Gaussian. A parabolic fit of the logarithmized data is finally used to obtain the parameters $N$ and $\sigma$. This procedure is repeated applying different shifts of the experimentally determined energies $E$ until the number of states with $E < 0$, i.e. the integral of the analytic function above from $E=\,-\infty$ to $E=\,0$, is equal to half the total number of states in the sample as found in our simulations. The extracted values of the fit depend on the borders that are chosen for the fit (in our case e.g. −100 to −70 meV for the exponential part) and therefore should be considered with care. Δ corresponds to an offset in the energy axis. All fit values are listed in Table S1 of the SI.

## Electronic supplementary material

Supplementary Information

Supplementary Movie 2

Supplementary Movie 1

## Data Availability

Data is available from the authors upon reasonable request.
