# Supplementary material for: Energy barriers at grain boundaries dominate charge carrier transport in an electron-conductive organic semiconductor
Source: Sci Rep. 2018 Oct 5;8:14868. doi: 10.1038/s41598-018-33308-y (PMC6173704; doi:10.1038/s41598-018-33308-y)
Supplement: Supplementary file 1 — Supplementary Information [file 41598_2018_33308_MOESM1_ESM.pdf]

Supplementary Information

Energy barriers at grain boundaries dominate charge carrier transport in an electron conductive organic semiconductor

I. Vladimirov[1,2]*, M. Kühn[1*,+], T. Geßner[1], F. May[1,2], R.T. Weitz[1,2,3,4,5,#]

[1] BASF SE, FET Systems, Carl-Bosch-Straße 38, 67056 Ludwigshafen, Germany
[2] InnovationLab GmbH, Speyerer Str. 4, 69115 Heidelberg, Germany
[3] Physics of Nanosystems, Faculty of Physics, Ludwig-Maximilians University, Amalienstr. 54, 80799 Munich, Germany
[4] Center for Nanoscience (CeNS), Ludwig-Maximilians University Munich, Schellingstr. 4 80799 Munich, Germany
[5] Nanosystems Initiative Munich (NIM), Schellingstr. 4 80799 Munich, Germany

* I.V. and M.K. contributed equally
+ email: michael.b.kuehn@basf.com
# email: thomas.weitz@lmu.de

Table of Contents

1.DETERMINATION OF THE DENSITY OF STATES (DOS) FROM VGS-EA MEASUREMENTS 3

2.DETAILS OF THE THEORETICAL REALIZATION OF THE THIN FILM MORPHOLOGY AND ENERGY LANDSCAPE 9

2.1.GENERATION OF THE THIN FILM MORPHOLOGY 9

2.2.CALCULATION OF HOPPING RATES 14

2.3.CALCULATION OF ELECTRON AFFINITIES AND ENERGY LANDSCAPE IN THE LUMO 15

2.4.CALCULATION OF THE DENSITY OF STATES (DOS) OF THE POLYCRYSTALLINE THIN FILMS 17

2.5.COMPARISON BETWEEN THE CALCULATED DOS AND THE EXPERIMENT 19

2.6.IMPACT OF THE TORSION OF THE MOLECULE ON THE DOS 22

2.7.IMPACT OF THE ANGLE BETWEEN TWO CRYSTALS AT THE GRAIN BOUNDARY ON THE VALLEYS 23

2.8.ENERGY LANDSCAPE IN THE HOMO 24

3.THEORETICAL DESCRIPTION OF CHARGE TRANSPORT 25

3.1.CALCULATION OF TRANSFER INTEGRALS 25

3.2.CHARGE TRANSPORT SIMULATIONS 26

REFERENCES 32

1. Determination of the density of states (DOS) from V_{GS}-E_a measurements

From the temperature dependent transfer curves (for an example see **Figure S1a**), we have extracted the temperature dependence of the linear charge carrier mobility μ_{lin}. Representative measurements of the temperature dependence of μ_{lin} for a sample deposited from DMP:toluene (1:3) solution are shown in the main text and for all samples measured in our study in **Figure S1 b-d**. They allow us to quantify the extent of energetic disorder in the crystal. The mobility in the linear regime ($\mu_{lin} = \frac{L}{C_{ox} W V_{DS}} (\frac{\partial I_D}{\partial V_{GS}})$ at V_{DS} = 1 V) was calculated for different gate voltages. All transfer curves have been corrected for the changing threshold voltage with temperature. We have then calculated the activation energy E_a following an Arrhenius approach for reaction rates via $\mu = \mu_0 \, exp(-\frac{E_a}{k_B T})$. Upon cooling the films down to cryogenic temperatures one can observe an activated behavior with an activation energy as low as E_a= 21.2 meV in the highest quality films. Since we have not varied the dielectric of our transistors, it is a priori unclear whether E_a is dominated by interactions of the charge carriers with the dielectric (Fröhlich polarons)[1], or if E_a is a consequence of the disorder within the crystal. From previous measurements by Hulea et al., performed on rubrene single crystals it is known[1], that the polaron binding energy in our device geometry (given that in our case the distance between the conductive channel and the dielectric is twice that in the case of Hulea et al.) should be around 20 meV. This is consistent with the lowest E_a measured in our best ordered samples. This in turn also implies, that in the case we measure an E_a larger than 20 meV it is dominated by other sources of disorder (e.g. grain boundaries) and not by Fröhlich polarons. In addition, using our solution deposition method we are able to tune the morphology of the crystal, and can measure E_a for a variety of sample qualities.

The resulting E_a versus V_{GS} plots are shown in **Figure S2a, c, e** for the various solvent mixtures. The $E_a(V_{GS})$ dependence allows to calculate the density of states (DOS) utilizing the incremental change of E_a with gate voltage via $N(E) = (C/q)[1/(dE_a/dV_{GS})]$, where C is the capacitance of the dielectric, q the electronic charge and E the energy measured from the LUMO. The resulting DOS $N(E)$ (**Figure S2b, d, f**) gives the number of electronic states per eV cm³ in the energetic tail extending from the LUMO into the bandgap (here normalized by the height of the organic semiconductor

of 1.7 nm and assuming that charges are only localized in the first monolayer of the organic semiconductor). We are aware, that this method of calculation of the DOS leads to an overestimation of the respective E_0 and σ, since for example the contact resistance or the dependence of the charge sheet thickness on the gate voltage is not considered [2,3]. However, we are in this study only interested in the relative trends between samples of different crystallinity, which should be consistent with the formulas used here.

One can then fit known functional forms to the DOS in order to extract characteristic energy scales. For energies close to the LUMO (small negative E), we have assumed a Gaussian shape for the density of states (DOS, ($N(E) = \mathrm{N} * \exp\left(-\frac{(E+\Delta)^2}{2\sigma^2}\right)$), where σ is the variance of the DOS, N_0 the total number of states and E the energy measured from the LUMO energy band edge. Theoretical works assume that Gaussian broadening stems from disorder within single grains [4]. At lower energies (higher negative E in our figures; disorder here is assumed to stem from the grain boundaries), we can fit our data better with an exponential $N(E) = \mathrm{N}_0 * \exp\left(\frac{E+\Delta}{E_0}\right)$). The data extracted from the fits are summarized in **Figure S4** and plotted against various experimental measurements. We observe a correlation between the threshold voltage and the slope of the E_0 of the DOS (**Figure S4e**).

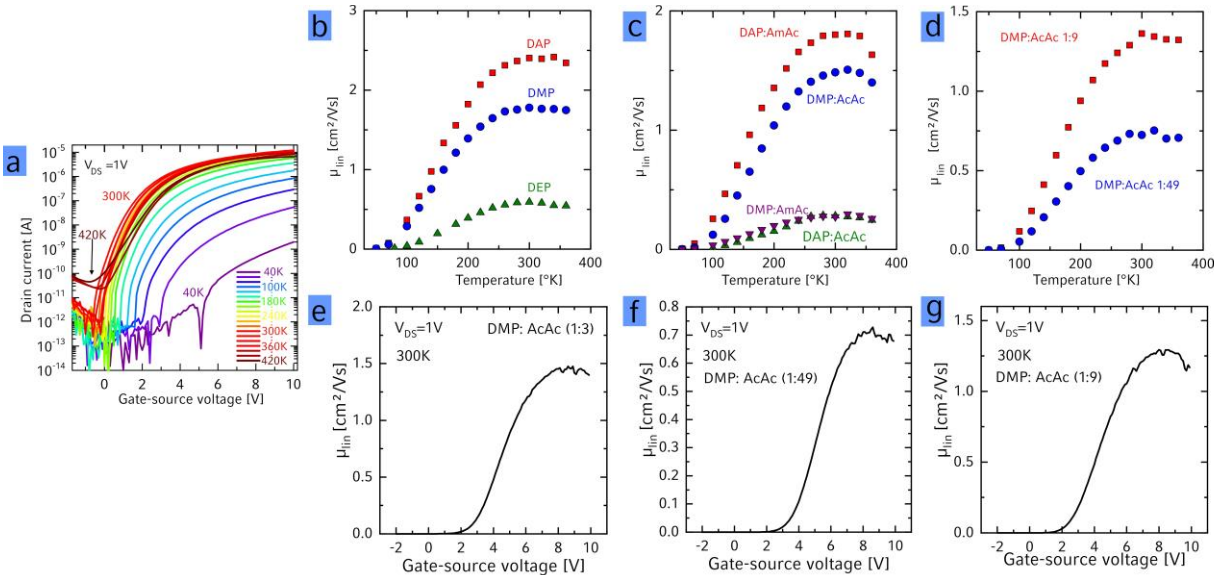

Figure S1: a) Example of temperature dependent transfer curves from which the temperature dependent mobility plot in Figure 1b of the main manuscript has been extracted. For this sample, the organic thin film was fabricated from a 1:3 mix between DMP and toluene. **b)-d)** Extracted temperature dependences of the linear mobility of the thin films investigated that were fabricated with the solvents or solvent mixtures indicated next to the curves. **e)-f)** Exemplary dependences of the linear charge carrier mobility on the gate voltage.

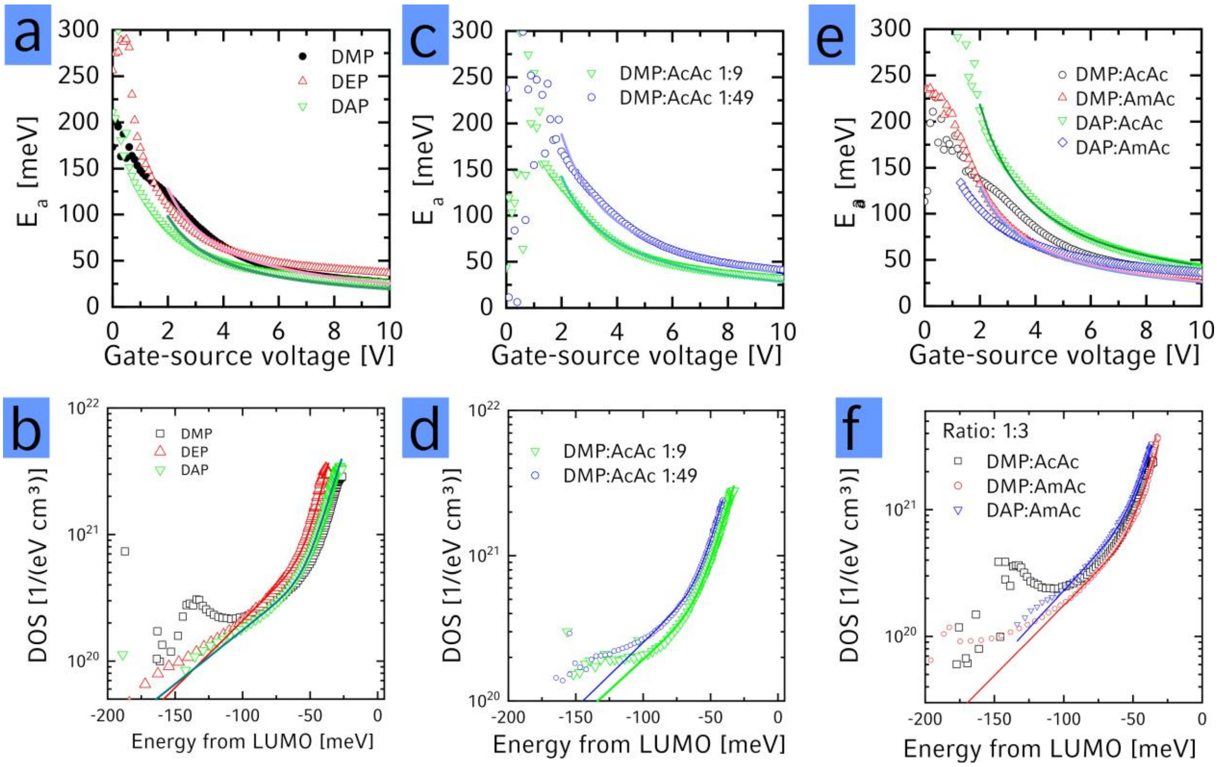

Figure S2: upper row (a,c,e): Activation energy E_a extracted from the temperature dependence of the mobility as described in the text for the different solvent mixtures. The fits are done according to equation 1 of the main manuscript. **Lower row (b,d,f):** DOS calculated from the respective E_a measurements for different solvent mixtures. The solid lines are fits to the combination of an exponential at low energies and a Gaussian density of states at energies close to 0 meV. The curves have been fit to the raw data as described in the methods section of the main manuscript. The extracted data are listed in **Table S1** and compared to other experimental values in **Figure S4**. We were not able to fit the data with the bump in the DOS at low energies (samples made from DMP and DMP:AcAc (1:3)).

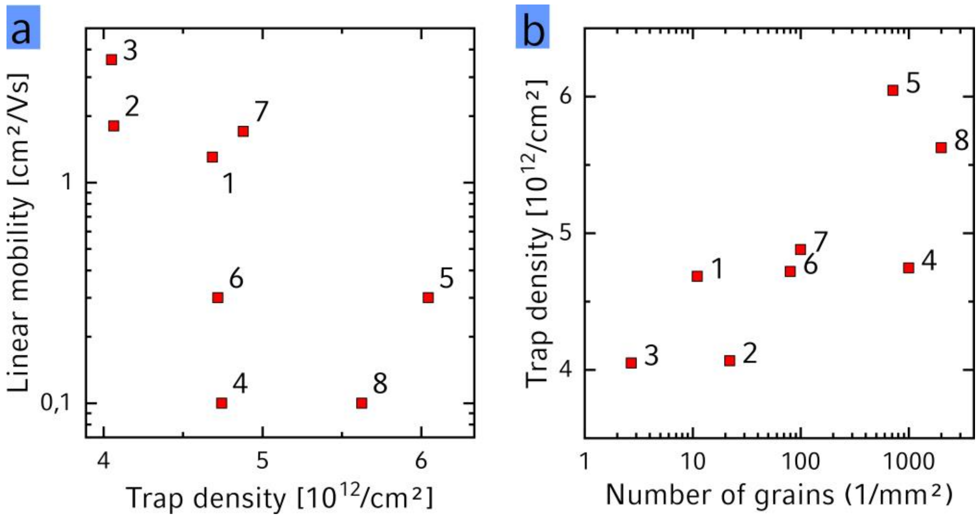

Figure S3: Comparison of trap densities extracted from the $E_a(V_{GS})$ plots shown in Figure 1d of the main manuscript and Figure S2 a), c) and e) to: **a)** the linear charge carrier mobility **b)** the number of grains extracted from optical measurements. The fits are described and the data is discussed in the main text.

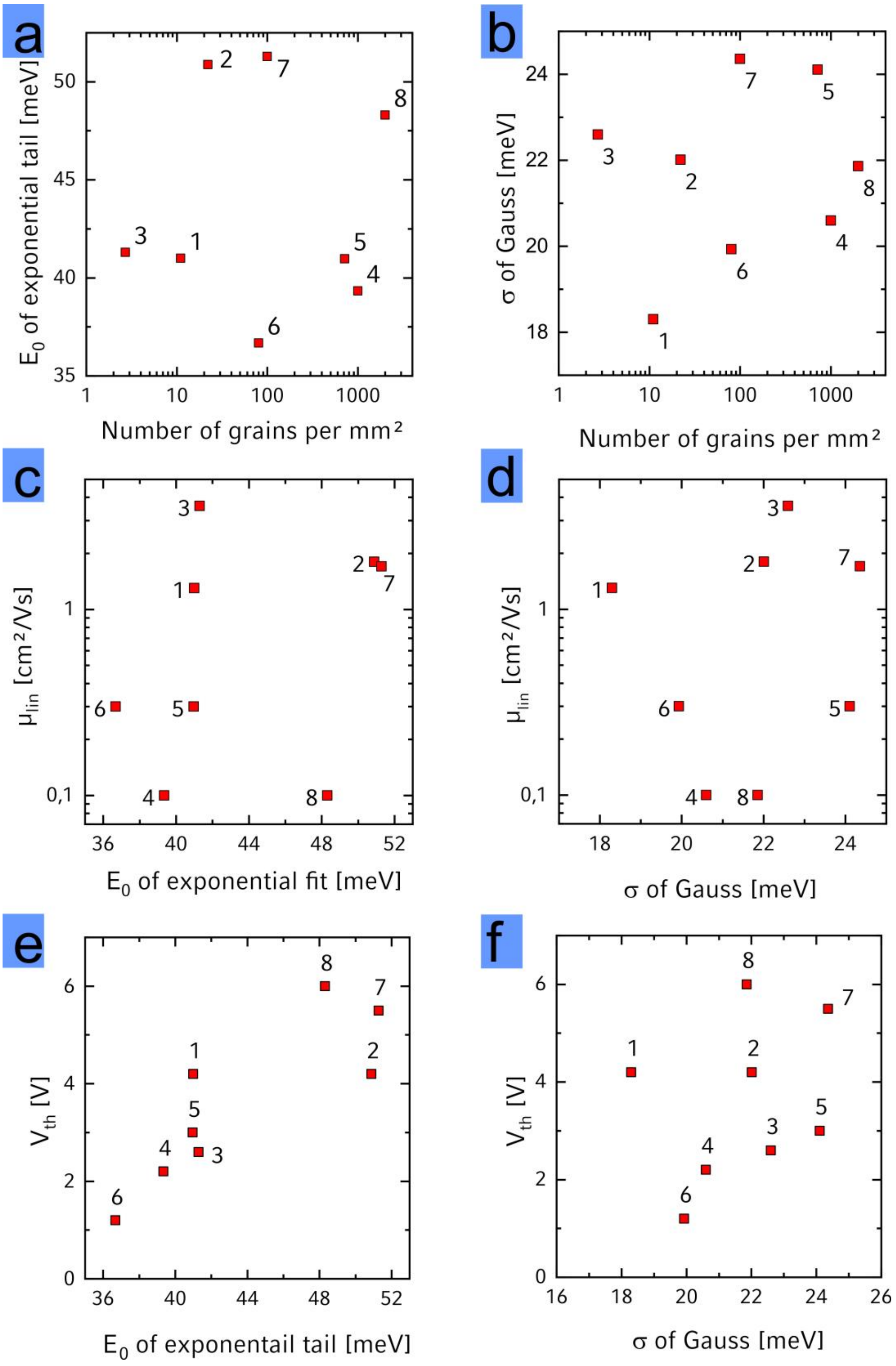

Figure S4: Comparison of the extracted slope of the exponential part of the and E_0 full width at half maximum (FWHM) σ as extracted from the fits in **Figure S2** in comparison with various experimental parameters. The solvents used for deposition of the semiconductor are: **1**: DEP, **2**: DAP, **3**:DMP:Tol (1:3), **4**: DMP:AmAc (1:3), **5**: DAP:AcAc (1:3), **6**: DAP:AmAc (1:3), **7**: DMP:AcAc (1:9), **8**: DMP:AcAc (1:49).

Solvent	Number of grains per mm²	μ_{lin}^{max}	μ_{lin}^{ave}	V_{th}^{ave}	$E_{a\ min}$	σ	N × 10^{20}	E_0	N_0x10^{20}	Δ
		[cm^2 /Vs]		[V]	[meV]		[$(eVcm^3)^{-1}$]	[meV]	[$(eVcm^3)^{-1}$]	meV
DMP	35	2.81	1.3 ± 0.6	4.2 ± 0.3	30.3±0.6	no good fit possible due to bump at low energies				
DEP	11	1.44	1.0 ± 0.2	4.2 ± 0.2	40.2±0.4	18.3	240.5	41	25.1	-2
DAP	22	2.74	1.8 ± 0.5	4.2 ± 0.3	30.9±0.4	22.01	190.3	50.9	16.84	-15
**mixture ratio**		**DMP:AcAc mixture**								
1:3	50	2.67	1.4 ± 0.6	5.8 ± 0.4	38.4±0.5	no good fit possible due to bump at low energies				
1:9	100	2.50	1.7 ± 0.4	5.5 ± 0.4	38.5±0.8	24.4	183.0	51.3	18.9	-17
1:49	2000	0.85	0.1 ± 0.1	6.0 ± 0.9	39.8±0.5	21.9	192.2	48.3	23.6	-8
	**Further ternary blends [1:3] ratio (all crystalized at 70 °C)**									
DMP:AmAc	1000	0.14	0.1 ± 0.0	2.2 ± 0.4	30.8 ± 0.2	20.6	209.4	39.3	30.2	-12
DAP:AcAc	715	0.31	0.3 ± 0.0	3.0 ± 0.7	45.9 ± 1.0	24.1	146.8	41.0	51.4	-20
DAP:AmAc	80	1.63	0.3 ± 0.3	1.2 ± 0.4	32.5 ± 1.1	19.9	190.1	36.7	41.3	-6
DMP:Toluene	2,7	4.42	3.6 ± 0.6	2.6 ± 0.1	21.2 ± 0.2	21.2	183.6	37.8	35.1	-17
DMP:NiEth		0.69	0.3 ± 0.3	0.8 ± 0.5	not measured					
DMP:oDCB		2.90	2.1 ± 0.5	1.7 ± 0.6	not measured					

Table S1: Overview of the electrical characteristics and fit values obtained.

2. Details of the theoretical description of the thin film morphology and energy landscape

2.1. Generation of the thin film morphology

2.1.1. PDI1MPCN2 molecule in the gas phase

The electronic ground-state structure of a single PDI1MPCN2 core (i.e. a single molecule without side chains see **Figure S5**), in the gas phase was optimized using density-functional theory (DFT) as implemented in the TURBOMOLE program package.[5] The hybrid functional B3LYP [6] was utilized in combination with polarized triple-ζ valence (def2-TZVP) basis sets.[7] The optimized structure reveals a twisted polycyclic core with a torsion angle of 16.4°, see **Figure S6**. The energy difference between the twisted and the corresponding untwisted (i.e. planar) structure is calculated to –54 meV. The dipole moment of the twisted core amounts to 1.70 Debye normal to the molecular plane due to the cyano groups both pointing behind the same side of the molecular plane (compared to 0.02 Debye for the untwisted structure), 1/3 of the trace of the quadrupole tensor is –160 a.u. (compared to –161 a.u.) with an anisotropy of 33 a.u. (compared to 36 a.u.). The electrostatic potential is shown in **Figure S5 (right)**. We note in passing, that a structure optimization with the generalized gradient approximation functional of Becke [8] and Perdew [9] (BP86) or application of the conductor-like screening model (COSMO) [10], that considers the crystal environment simply by assuming a dielectric continuum of relative permittivity ε_{r} = 3, leads to a very similar twisted core structure. With regard to charge transport, torsion can lead to closer packing, as observed in PDI-FCN$_2$.[11] At the same time torsion can reduce thermal molecular fluctuations in the crystal. This is illustrated in **Figure S6,** where the difference in total energies between a cofacial dimer with twisted cores (upper right-hand corner) and a cofacial dimer with planar cores (lower right-hand corner) is plotted against the shift (Δz) along the long molecular axis (B3LYP/TZVP with Grimme's dispersion correction [12]). The steep increase in this dimer interaction energy difference for increasing shifts along the long molecular axis means that thermal fluctuations are energetically more unfavorable for the twisted cores than for the planar cores due to the intercalation in packing that prevents sliding. The reduction of thermal fluctuations leads to more robust transfer integrals which in turn lead to larger charge carrier mobilities.[13] Furthermore also energetic disorder within the crystal grains can be minimized due to reduced thermal fluctuations which also increases mobilities.

For the electron affinity (EA) in the solid state crystal we again use the COSMO method to include the polarization stabilization of the surrounding, which shifts the gas-phase EA of 3.310 eV to EA = 3.866 eV (both for B3LYP/def2-TZVP). Note that the cyano groups are necessary

in order to deepen the EA of the unsubstituted core by 0.515 eV (EA = 3.351 eV for the bare core via COSMO, B3LYP/def2-TZVP), as this improves the electron injection from the gold contacts as well as it increases the air stability of the molecule.

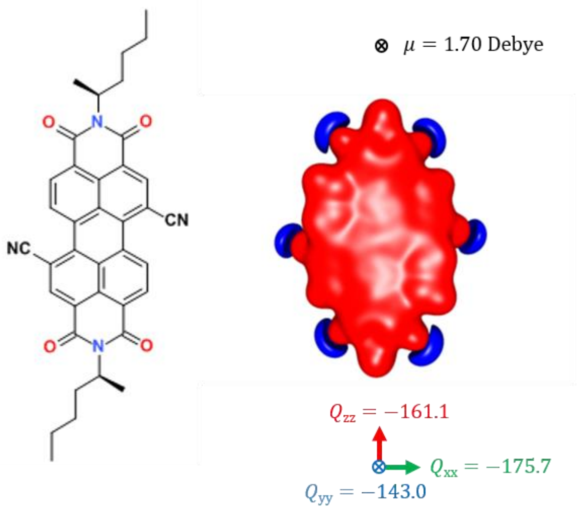

Figure S5: Chemical structure of PDI1MPCN2 (with side chains), N,N´-di((S)-1-methylpentyl)-1,7(6)-dicyano-perylene-3,4:9,10-bis(dicarboximide), and electrostatic potential of the PDI1MPCN2 core. Isosurfaces are drawn blue (–1 V) and red (1 V). Quadrupole moments are given in atomic units along all three principal axes. The dipole moment is normal to the molecular plane due to the torsion of the core with cyano groups pointing behind this plane.

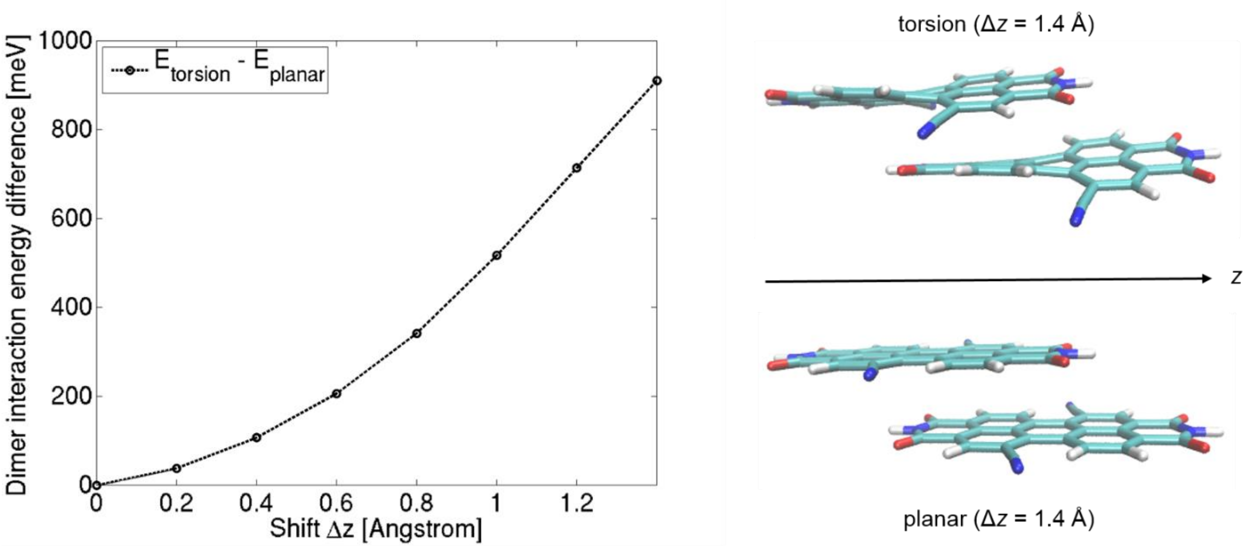

Figure S6: Difference in total energies between a dimer with twisted PDI1MPCN2 cores (upper right-hand corner) and a dimer with planar cores (lower right-hand corner) plotted against the shift (Δ*z*) along the long molecular axis. The difference in total energies in case of no shift (Δ*z* = 0) is set to zero. The two molecules forming a dimer are oriented in a cofacial manner with a constant distance of 3.2 Å normal to the molecular plane. B3LYP/TZVP is used along with Grimme's dispersion correction.[12]

2.1.2. Generation of perfectly crystalline monolayer morphology

Perfectly crystalline monolayer morphologies were generated based on the optimized molecular structure described above in combination with the experimentally determined triclinic lattice parameters (a = 5.46 Å, b = 8.54 Å, c = 17.45 Å, α = 89.98°, β = 99.35°, γ = 112.71°).

Since the orientation of the PDI1MPCN2 molecule in its unit cell was not accessible in the experiment, we have tried out different orientations defined by three angles (θ,ϕ,Ψ). First, the same orientation as found experimentally for PDI-FCN_2 was assumed.[11] To this end the molecule was placed in a unit cell (a-direction along x-axis and b-direction in x-y-plane) with the center of mass in the origin, the long molecular axis along the z-axis and the normal along the x-axis. Then, the long molecular axis was tilted by θ = 30° around the x-axis and by ϕ = 260° (azimuthal angle) around the z-axis. Finally, the molecule was rotated around its long axis by Ψ = 100°. The resulting morphology reveals a ratio of the largest transfer integral (in a-direction) to the second largest transfer integral (in a+b-direction) of J_a/J_{a+b} = 366 which would lead to highly non-isotropic charge transport. We display the mobility tensor based on Jortner hopping rates for electric field directions within the transport plane from 0° to 180° in steps of 10° with respect to the x-axis (a-direction), see **Figure S20** (right). For details concerning the charge transport simulations see below. As the orientation of the sample within the OFET channel is random we average over all values and obtain a mean mobility of 2.92 cm^2/Vs together with a large standard deviation of 2.08 cm^2/Vs amounting to 71 % of the average value, see **Figure S19** (magenta data point on the left).

As a comparison, the experiment e.g. for highest degree of crystallinity as found for DMP:toluene (1:3) with a statistics of 130 transistors, 84 of which were measured on the same substrate, yields $\mu = 2.9 \pm 0.5$ cm^2/Vs indicating a much lower standard deviation of only 17 %. This experimental standard deviation has to be seen as an upper limit for our calculated standard deviations, since in experiment additional sources of error are included besides the bare averaging over different electric field directions. In contrast to this experimentally measured upper limit of 17 %, in our simulation a significantly larger standard deviation of 71 % is obtained, which is due to the strong dependence of the mobility on the direction of the applied electric field in case of an effective one-dimensional charge transport.

From the above comparison with the experimental standard deviation and the observation that the drop in the simulated mobility when introducing grain boundaries is much stronger than expected from the experiment, see **Figure S19**, we rule out an effective one-dimensional charge transport and propose another crystal structure. For this, we generated all possible orientations of the PDI1MPCN2 molecule in its unit cell, i.e. we systematically scanned all three angels θ, ϕ and Ψ as defined above in steps of 1°, under the following conditions: (i) The polar

angle θ is restricted to be between 30° and 45°, since in the experiment it is determined to θ = 40 ± 7°, calculated from the length of the molecule (2.4 nm) and the height of a monolayer found in experiment (d = 1.8 ± 0.2 nm). (ii) The minimal distance between atoms belonging to neighboring PDI1MPCN2 cores is estimated to be 2.5 Å. This criterion mainly concerns distances between neighboring C–N-groups, since they are sticking out of the molecular plane. For the side chains the minimal distance criterion between atoms was loosened to 1.5 Å since they are more flexible than the cores and therefore expected to relax for every reasonable molecular arrangement. Taking into account both conditions, (i) and (ii), the simulated mobility in a perfect crystal with the standard deviation closest to the experimental result of 17 % (DMP:toluene (1:3)) is found for angles θ = 30°, ϕ = 190°, Ψ = 0°, see **Figure S7**. As expected, the resulting morphology reveals an effective two-dimensional charge transport with a transfer integral ratio of J_a/J_{a+b} = 1.4. The simulated mobility tensor is shown in **Figure S20** (left) and reveals a significantly smaller anisotropy than in case of an effective one-dimensional charge transport (right). The corresponding mean mobility amounts to 25.13 cm^2/Vs with a standard deviation of 5.86 cm^2/Vs (23 %), see **Figure S19** (cyan data point on the left), such that we now have achieved a good agreement with the experimental standard deviation. We note that the absolute values of our simulated mobilities cannot be compared directly to the corresponding measured mobilities, as described below.

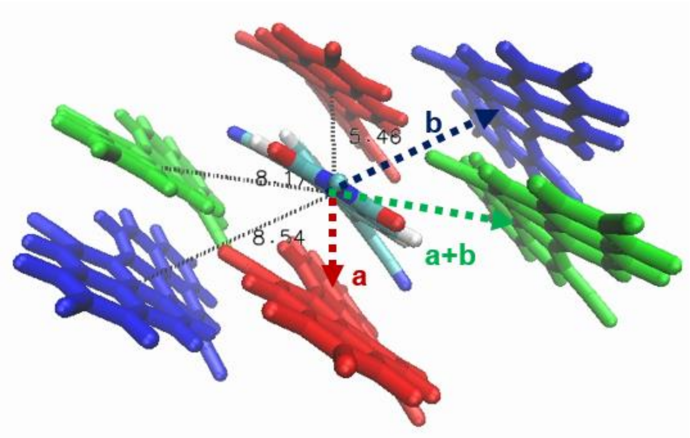

Figure S7: Molecular arrangement of the PDI1MPCN2 core within the unit cell assuming a two-dimensional charge transport (J_a/J_{a+b} = 1.4, θ = 30°, ϕ = 190°, Ψ = 0°). The *a*-, *b*- and *a+b*-directions are also drawn with distances in Å.

2.1.3. Realization of polycrystalline morphologies

In order to study the dependence of the charge transport on the degree of crystallinity, monolayers were generated in a 100 nm x 100 nm box containing 2x2, 5x5, 10x10 and 15x15 crystal grains assuming a two-dimensional charge transport within the crystal grains (J_a/J_{a+b} = 1.4, θ = 30°, ϕ = 190°, Ψ = 0°), see lower right-hand pictures in **Figures S8 – S10**. The total number of molecules in the generated morphologies are 22.979 (2x2), 21.933 (5x5), 20.381 (10x10) and 18.987 (15x15). Additionally, corresponding morphologies were generated assuming a one-dimensional charge transport within the crystal grains (J_a/J_{a+b} = 366, θ = 30°, ϕ = 260°, Ψ = 100°). In all cases equidistant seeding points were chosen and radial grain growth with the same speed within all crystal grains is assumed, see also Supplementary **Movie S1**. The *a*-direction of each crystal grain is oriented randomly. Molecules are only generated if the distance of atoms belonging to neighboring grains is larger than 2 Å. Relaxation of the side chains of the molecules at the grain boundaries was taken into account implicitly by shortening the side chains to isopropyl and thus allowing the (twisted) cores at the grain boundaries to come closer while at least partially considering the steric effect of the side chains.

2.2. Calculation of hopping Rates

The theoretical description of charge transport in perfectly crystalline organic semiconductors, such as OFETs, remains a challenge. In contrast to disordered amorphous organic semiconductors, where the hopping model of localized charge carriers delivers an appropriate description of the microscopic processes,[14] in perfectly crystalline organic semiconductors also delocalized band-like transport and transport in an intermediate regime called transient localization are subject to ongoing discussions.[4,13] In the case of polycrystalline morphologies, as present in our study, charge transport is mainly limited by the presence of grain boundaries. Due to small electronic couplings across those grain boundaries (average transfer integral of $J \approx 1$ meV compared to $J \approx 100$ meV within the crystal grains) in combination with a large energetic disorder, a delocalization of the charge carrier over neighboring crystal grains is unlikely and thus application of the hopping model is appropriate.

In a recent charge transport study on a very similar n-type organic semiconductor, Cl_2-NDI, the Levich-Jortner hopping model [15] was successfully applied.[4] In contrast to the more commonly known (classical) Marcus hopping model[16] intramolecular modes are treated quantum-mechanically within Levich-Jortner hopping theory, which is more appropriate at ambient conditions (where typically for C–C bond stretches $\hbar\omega^{\mathrm{int}} \approx 0.2\,\mathrm{eV} \gg k_B T$) than a classical treatment. As a result, for Cl_2-NDI the simulated temperature dependence of the mobility tensor was found to be in significantly better agreement with the experimental findings in case of the Levich-Jortner hopping model. The corresponding rate expression for a hop between two molecules *i* and *j* reads

$$k_{ij} = \frac{2\pi}{\hbar} \frac{|J_{ij}|^2}{\sqrt{4\pi\lambda^{\mathrm{out}} k_B T}} \sum_{N=0}^{\infty} \frac{S^N \exp(-S)}{N!} \exp\left\{-\frac{[\Delta E_{ij} - N\hbar\omega^{\mathrm{int}} - \lambda^{\mathrm{out}}]^2}{4\lambda^{\mathrm{out}} k_B T}\right\} \quad \textbf{Eq.1}$$

The site energy differences ΔE_{ij} (energy landscapes are shown in **Figures S8-S10**) and the transfer integrals J_{ij}, which have to be evaluated for all molecular pairs *ij,* mainly determine the charge transport characteristics across grain boundaries, see below. It is assumed that the donor is initially in its vibrational ground state, while *N* describes all excited vibrational states of the acceptor that are separated by $\hbar\omega^{\mathrm{int}} = 0.2\,\mathrm{eV}$ (see above), with $S = \lambda^{\mathrm{int}}/\hbar\omega^{\mathrm{int}}$ being the Huang-Rhys factor and $k_B T$ the thermal energy with $T = 300\,\mathrm{K}$. The intramolecular reorganization energy λ^{int} accounts for the fast relaxation of the nuclear coordinates upon charging a molecule. In case of PDI1MPCN2 it is calculated to $\lambda^{\mathrm{int}} = 0.23\,\mathrm{eV}$ at the B3LYP/def2-TZVP level in the gas phase. The outer-sphere reorganization energy λ^{out} accounts for the slow relaxation of the surrounding molecules after the charge-transfer process

has taken place and is chosen to amount to $\lambda^{\text{out}} = 0.05\,\text{eV}$ as in Cl_2-NDI.[4] If not stated otherwise, energies *E* and transfer integrals *J* are only discussed for the two-dimensional charge transport morphologies (J_a/J_{a+b} = 1.4, θ = 30°, ϕ = 190°, Ψ = 0°) in the following.

2.3. Calculation of electron affinities and energy landscape of the LUMO

Site energies, i.e. the electron affinities (EAs) in the solid state, for molecule *i*,

$$E_i = \text{EA}_{\text{COSMO}} + \frac{1}{4\pi\varepsilon_0}\sum_{a_i}\sum_{\substack{b_k \\ k\neq i}} \frac{\left(q_{a_i}^{(-1)} - q_{a_i}^{(0)}\right) q_{b_k}^{(0)}}{\varepsilon_\text{r}\,\left|\boldsymbol{r}_{a_i} - \boldsymbol{r}_{b_k}\right|} \qquad \textbf{Eq.2}$$

were calculated by combining the solid-state EA from DFT via the COSMO method including polarization-stabilization, EA_{COSMO}, (which is identical for all molecules) with the electrostatic (Coulomb) energy difference between the charged (-1) and the neutral (0) molecule *i* interacting with all neighboring molecules *k* within a cutoff of 8 nm between the molecular center of masses.[17] $q_{a_i}^{(0)}$ describes the partial charges of all atoms *a* belonging to neutral (0) molecule *i,* which were taken from Merz-Kollman fits of the electron density in the gas phase at the B3LYP/def2-TZVP level. A relative permittivity of ε_r = 3 was used. As only energy differences enter **Equation 1**, we give all site energies with respect to the peak in the energy distribution (see also **Figures S11** and **S12**) representing the EA in the center of a crystal grain (here: 4.038 eV). We note that the commonly used label „Energy from LUMO“ is somewhat misleading, since all energies are plotted with respect to *electron affinity* in the solid state in the center of a crystal grain. The resulting energy landscapes reveal valleys, i.e. trap states, (energy < 0) as well as barriers (energy > 0) at the grain boundaries for all studied morphologies, see **Figures S8 – S10**.

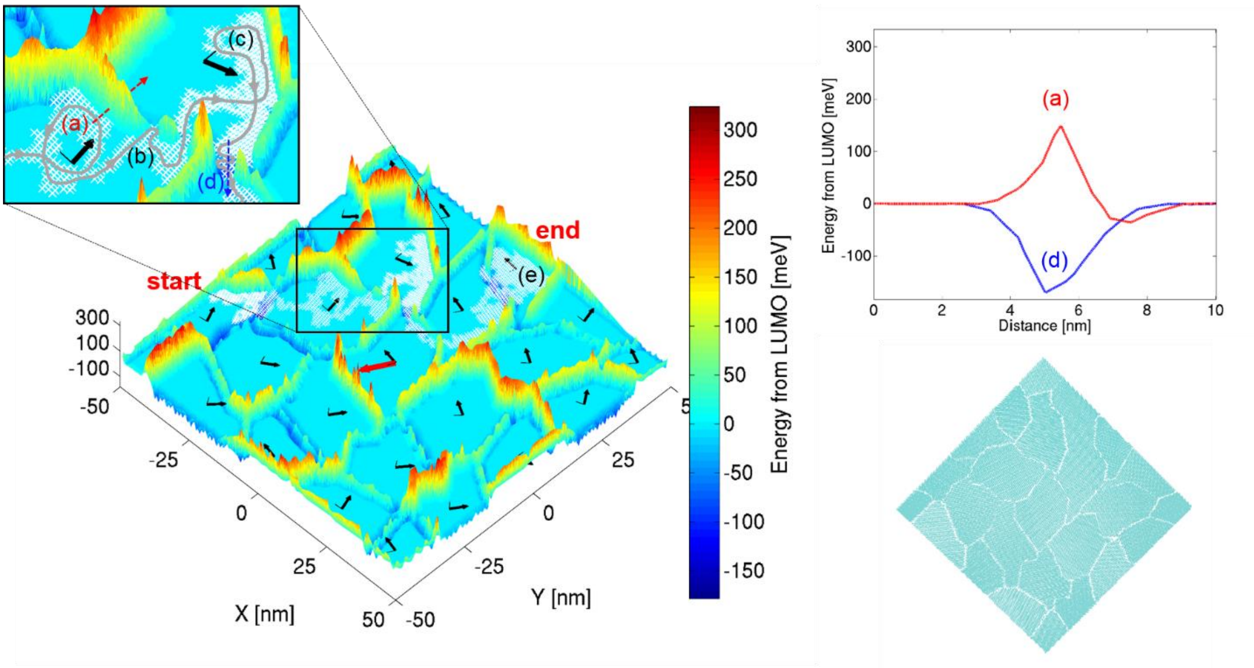

Figure S8: Electrostatic energy landscape for one electron (i.e. the EA) in a monolayer of 5x5 crystal grains per $(100nm)^2$ reveals valleys (dark blue) as well as barriers (red) (see also main text). The black arrows indicate the *a*-directions within the crystal grains, whereas short black lines indicate the *a*+*b*-directions. The underlying morphology is shown in the lower right-hand corner. An example of a valley and a barrier are extracted in the top right graph. A typical electron trajectory between "start" and "end" with mobility μ = 1.06 cm^2/Vs is drawn (white mesh and grey line). The white mesh characterizes all molecules that are occupied by the charge carrier at least once during the total simulation time of 70 ps. The electron mainly travels against the applied electric field (field strength F = 1V/100nm) visualized by the red arrow as described in the main text.

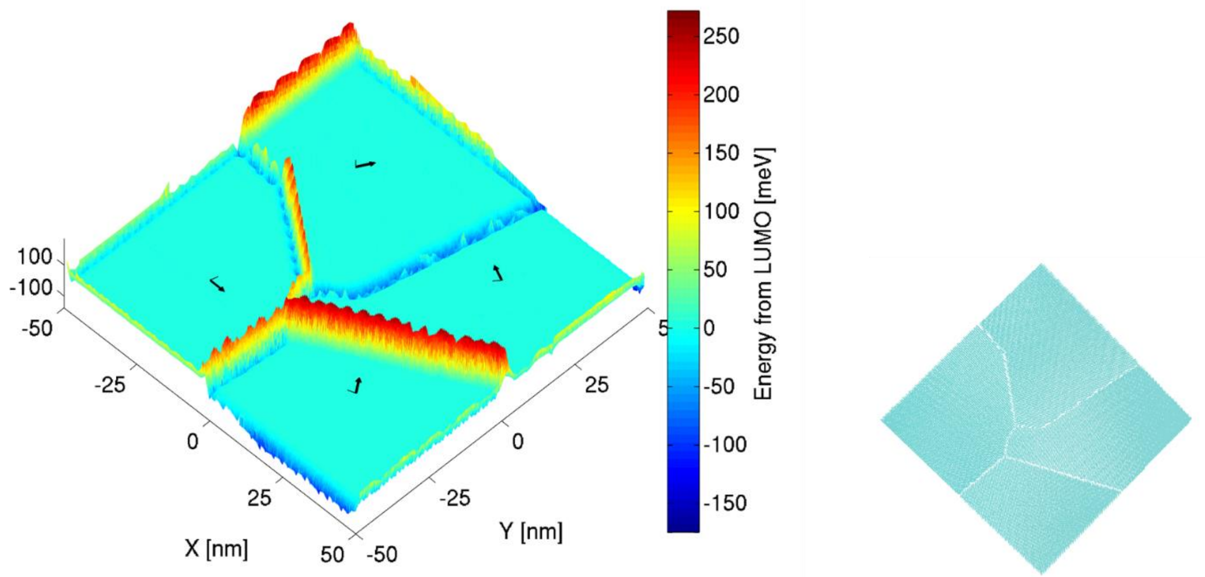

Figure S9: Electrostatic energy landscape in a monolayer of 2x2 crystal grains per $(100nm)^2$. For further details see **Figure S8**.

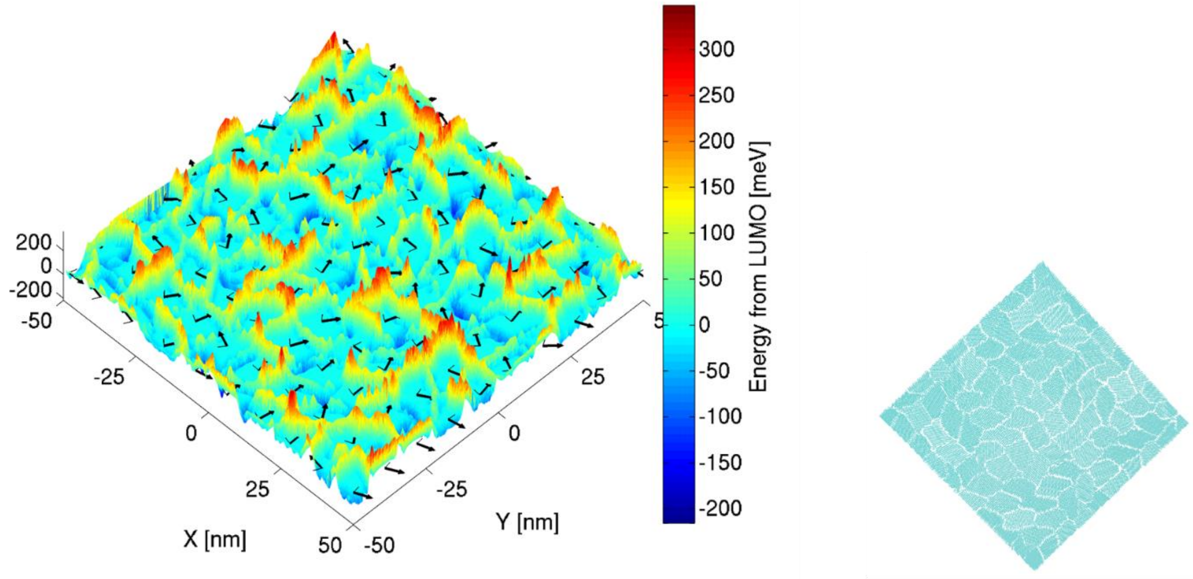

Figure S10: Electrostatic energy landscape in a monolayer of 10x10 crystal grains per $(100nm)^2$. For further details see **Figure S8**.

2.4. Calculation of the density of states (DOS) of the polycrystalline thin films

The density of states (DOS), which is given by the number of states per energy interval divided by the volume of our monolayers (100 x 100 x 1.8 nm^3, with the height of 1.8 nm taken from the experiment), is shown in **Figure S11**. To improve statistics, two realizations of grain formation (random *a*-directions) are taken into account for each *N*x*N* morphology (*N* = 2, 5, 10). For the 10x10 morphology we have checked that the DOS remains virtually unchanged when four realizations of grain formation are taken into account. The DOS, which is only due to static disorder caused by the presence of grain boundaries in our study, is found to be non-symmetric. The standard deviation (STDEV) of the DOS, i.e. the disorder, increases with increasing *N* from STDEV(2x2) = 27 meV via STDEV(5x5) = 43 meV through to STDEV(10x10) = 61 meV, see **Figure S11**. There are roughly the same number of states on both sides of the peak, i.e. there are as many valleys (energy < 0) as there are barriers (energy > 0) for all studied morphologies. We note that the choice of ε_r = 3 in **Equation 2** often leads to an overestimation of the screening of the additional charge as compared to a microscopic approach taking into account the polarizability self-consistently on atomic level via the Thole model.[17] However, as the computation of polarization response for several realizations of

large-scale morphologies is computationally demanding and requires well-equilibrated grain boundaries, we stick to **Equation 2** but suggest that the whole range of values between ε_r = 3 and ε_r = 1 should be considered.

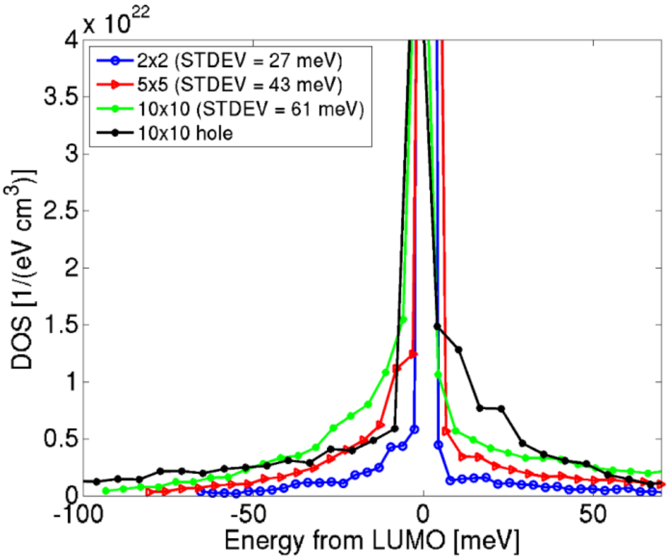

Figure S11: Density of states (DOS) for electrostatic energies of one electron (i.e. electron affinities, EA) in a monolayer of *N*x*N* crystal grains (*N* = 2, 5, 10) per (100nm)^2 with the vertical axis plotted on a non-logarithmic scale. The standard deviation (STDEV) of the DOS, i.e. the disorder, increases with *N*. Additionally, the DOS is shown for electrostatic energies of one hole (i.e. ionization potentials) for the case of 10x10 crystal grains per (100nm)^2. For further details see **Figure S12**.

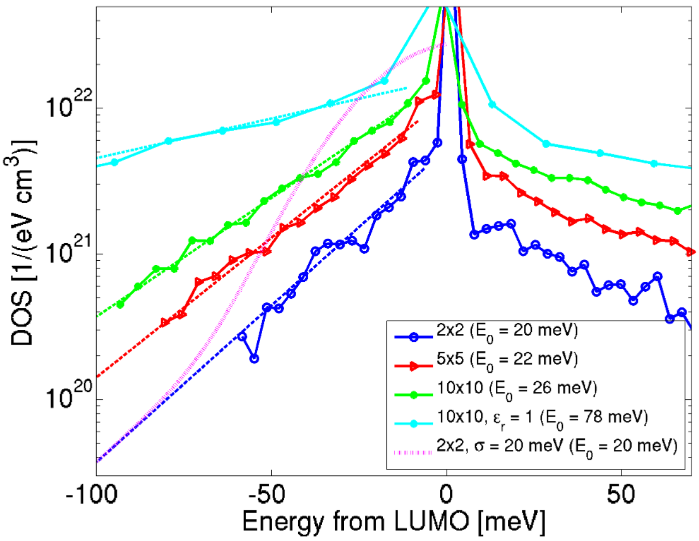

Figure S12: Density of states (DOS) for electrostatic energies of one electron (i.e. electron affinities, EA) in a morphology of *N*x*N* crystal grains (*N* = 2, 5, 10) per (100nm)^2. An exponential decay ($N_{\text{exp}}\, e^{E/E_0}$) of the trap states (energy < 0) due to grain boundaries is observed. Corresponding fits are drawn using dashed lines with E_0(2x2) = 20 (±2) meV, E_0(5x5) = 22 (±2) meV and E_0(10x10) = 26 (±3) meV. For 10x10 we compare to ε_r = 1 with a three-fold slower exponential decay due to reduced screening. Additionally, a Gaussian broadening of the DOS peak with standard deviation σ = 20 meV due to thermal fluctuations could be assumed keeping the normalization constant. The resulting DOS ($N_{\text{exp}}\, e^{E/E_0} + N_{\text{Gauss}}\, e^{-E^2/2\sigma^2}$) is plotted for the 2x2 crystal grains morphology using dotted lines.

In order to compare to the experiment, in **Figure S12** we display the DOS logarithmically which reveals a mono-exponential decay ($N_{\mathrm{exp}}\, e^{E/\,E_0}$) of the trap states (energy $E < 0$) due to grain boundaries which is absent for E > 0, see also **Figure S11**. For the traps, E_0 increases (slower exponential decay) with the number of crystal grains per (100nm)^2 from E_0(2x2) = 20 (±2) meV via E_0(5x5) = 22 (±2) meV and E_0(10x10) = 26 (±3) meV through to E_0(15x15) = 28 (±3) meV, see **Figure S13**. The observed trend remains stable and the calculated values remain within the given errors when changing the bin width of the underlying histogram. We attribute the observed increase of E_0 to a higher degree of irregularity in the grains which can be best seen by comparing the 2x2 to the 10x10 morphology, see **Figures S9** and **S10** and **Figures 3a and 3b** in the main paper. In other words: the sharper edges at the grain boundaries occurring for highly irregular shapes lead to deeper traps. As shown above, assuming ε_{r} = 3 overstimates the screening, so we also consider the other extreme case of ε_{r} = 1 which would triple all values for E_0.

2.5. Comparison between the calculated DOS and the experiment

As discussed in the main text the experimentally determined values of the DOS should be handled with care. Nevertheless we compare the extracted characteristics to the obtained theoretical values. The resulting range of values for E_0 agrees well with experiment, where E_0 = 37–51 meV is found for the deep trap states which are also exponentially distributed, see **Figure S13**. However, a clear comparison between theory and experiment is difficult since in the case of some solvents the value obtained for the experimental E_0 strongly depends on the energy interval chosen for fitting the exponential decay (in **Figure S13** the experimental E_0 are obtained by fitting an exponential decay between −100 meV and −70 meV).

Additionally, one could assume a Gaussian broadening of the DOS peak with standard deviation σ, which is due to dynamic disorder caused by thermal fluctuations within the crystal grains. The resulting DOS ($N_{\mathrm{exp}}\, e^{E/\,E_0} + N_{\mathrm{Gauss}}\, e^{-\,E^2/\,2\,\sigma^2}$) is plotted for the 2x2 morphology keeping the normalization of the original data for E < 0 using σ = 20 meV in agreement with the experiment. Note that relaxation of our morphologies, e.g. by molecular dynamics simulations, would be able to reproduce this Gaussian DOS with σ ≈ 20 meV due to the thermal fluctuations of the molecules as shown for a naphthalene diimide.[4] However, such an approach would mask the exponential decay visible for the less probable deep trap states as the Gaussian completely covers the tail states in **Figure S12**.

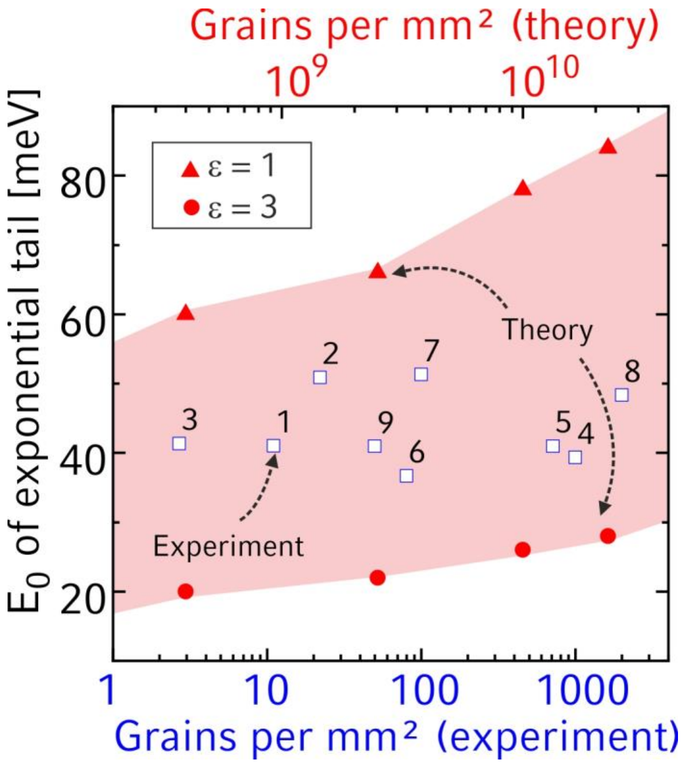

Figure S13: E_0 of the exponential decay of the trap states (energy $E < 0$) in the DOS shown in **Figure S2** and **S4** within a monolayer of NxN crystal grains (N = 2, 5, 10, 15) per $(100\text{nm})^2$ for ε_r in the range between 3 and 1 (red data points). Experimental E_0 are also shown (blue data points) for different solvents: **1**: DEP, **2**: DAP, **3**:DMP:Tol (1:3), **4**: DMP:AmAc (1:3), **5**: DAP:AcAc (1:3), **6**: DAP:AmAc (1:3), **7**: DMP:AcAc (1:9), **8**: DMP:AcAc (1:49). **9:** DMP:AcAc (1:3),

An estimate for the threshold charge carrier density can also be extracted from the calculated DOS. It is assumed that the charge transport (beyond the threshold voltage / threshold charge carrier density) starts at energies where the Gaussian broadening of the DOS peak begins to dominate over the exponential decay, i.e. at energies where all deep trap states are filled, see **Figure S12**. By occupying each state with a charge carrier up to this threshold energy, i.e. integrating the DOS from energy = $-\infty$ to the threshold energy, a threshold charge carrier density can be calculated. In **Figure S14 (left)** the threshold charge carrier densities are plotted for threshold energies of –30, –50 and –70 meV. We suggest a threshold energy of –70 meV to yield the best description for our samples as this is the energy, where the exponential tail becomes visible under the Gaussian, see **Figure S12**. As expected, the threshold charge carrier density increases with the number of crystal grains per area, since the number of exponential (deep) trap states (that need to be filled with charge carriers) also increases.

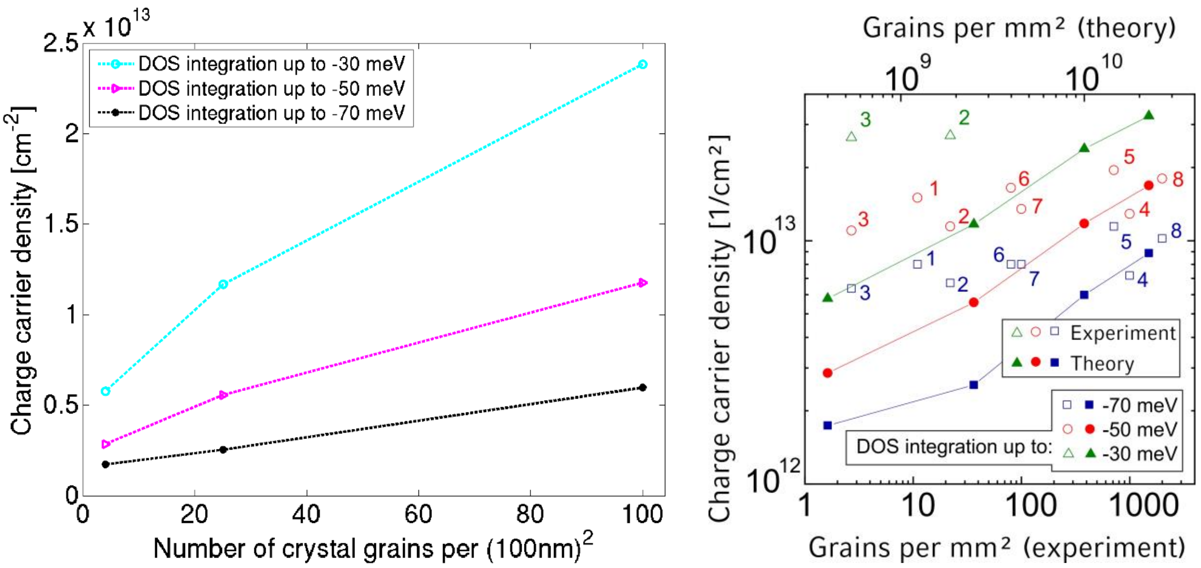

Figure S14: Threshold charge carrier densities within a monolayer of *N*x*N* crystal grains (*N* = 2, 5, 10) per (100nm)^2 calculated by integrating the DOS, see **Figure S12**, from energy = $-\infty$ to energy = -30, -50 and -70 meV. **(right)** Charge carrier density in the monolayer for filling all trap states up to cutoff energies of –30/50/70meV (green/red/blue) as obtained from DOS integration from theory (full symbols) and experiment (open symbols). Solvents used in the experiment are abbreviated the same as in **Figure 2** of the main manuscript (**1**: DEP; **2**: DAP; **3**:DMP:Tol1:3; **4**:DMP:AmAc 1:3; **5**: DAP:AcAc 1:3; **6**: DAP:AmAc 1:3; **7**: DMP:AcAc 1:9; **8**: DMP:AcAc 1:49).

Furthermore we have compared these densities to experimental values in **Figure S14 (right)** As one can see, the integrated densities from the experimental data agree with the trend obtained from theoretical calculations. In **Figure S14** it is also obvious, that the grain density extracted from experiment and assumed in theory is not the same. On the one hand, as we have employed ab-initio simulations at atomistic resolution, the regime for the degree of crystallinity we could cover considering roughly 20.000 molecules ranges from $4x10^8$ to $2.25x10^9$ grains per mm^2 in theory. On the other hand, crystal sizes determined by optical microscopy could be significantly smaller than the real grain size due to limited resolution (e.g. since also translational grain boundaries cannot be seen with polarization microscopy). Second, the simulations assume perfect crystalline order within the grains and only consider disorder in the film at the grain boundaries. This situation is however usually not found in experiments, where further traps might emerge for example from substrate roughness or impurities. Note that in addition, relaxation of our morphologies, e.g. by molecular dynamics simulations, would symmetrically broaden the sharp peak at E = 0 reproducing also the Gaussian part of the DOS with $\sigma \approx 20$meV due to the thermal fluctuations of the molecules [4]. However, due to insufficient statistics resulting from the ab-initio approach, such a relaxation would mask the exponential decay originating from the grain boundaries which is in the focus of our investigation (see below).

2.6. Impact of the torsion of the molecule on the DOS

To learn more about the impact of the molecular structure itself on the DOS, we have investigated the influence of the torsion in the molecular plane which is responsible for the dipole moment of 1.70 Debye. To this end we have taken one 10x10 morphology obtained for the twisted core structure and compared the energies to the same morphology but mapping the planar core onto the originally twisted core and then using the partial charges for the planar molecule (dipole moment of 0.02 Debye) in **Equation 2** to evaluate the energies. Comparing the original DOS to the DOS that would result from a planar core in **Figure S15 (left)** we conclude that indeed the torsion and the resulting dipole moment is the source for deep valleys and high barriers. The energy landscape is much smoother in case of the planar core with lower E_0 = 14 meV for the valleys (versus 26 meV in case of the torsion) and smaller standard deviation of 40 meV (versus 61 meV). The smaller standard deviation is also visible in the correlation plot in **Figure S15 (right)** where due to the similar morphology all energies are slightly correlated.

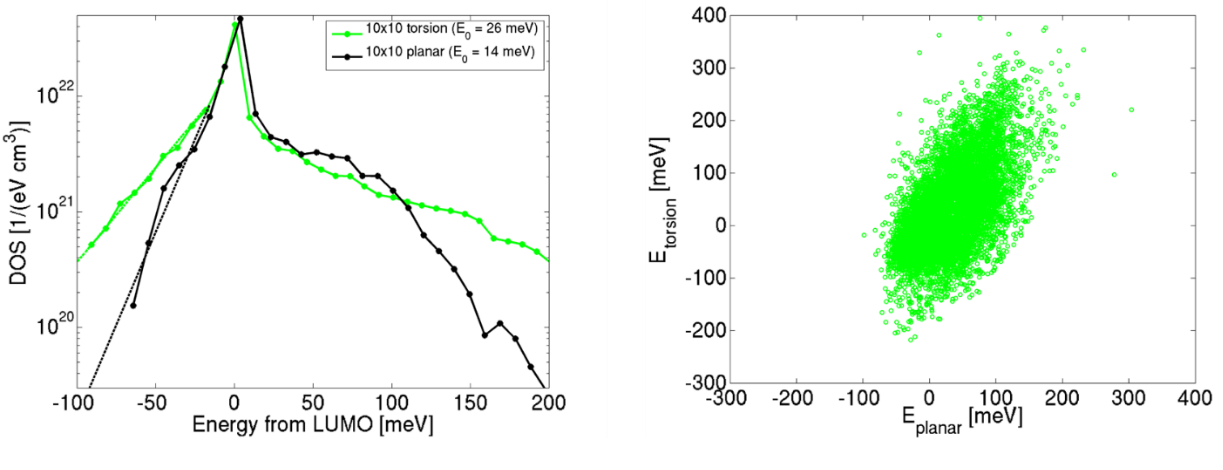

Figure S15: Comparison of the DOS for a 10x10 morphology for twisted (green) versus planer cores (black). The dipole moment due to the torsion of the twisted core leads to deeper energy traps and higher activation barriers. On the left-hand side it is shown that there is a correlation between the energies as both DOS were obtained based on a similar morphology by mapping the planar cores on the originally twisted cores (see text).

2.7. Impact of the angle between two crystals at the grain boundary on the valleys

To gain insight into the impact of the angle between different grain boundaries, we have also performed simulations. In detail, we have investigated the correlation between the relative angles of the *a*-directions belonging to two neighboring crystal grains and the mean energy of the resulting grain boundary. First, for each of the four realizations of the 10x10 morphology all molecules from a certain crystal grain were labelled by the corresponding angle of the *a*-direction relative to the *x*-axis. Second, all molecular pairs constituting grain boundaries were determined by checking whether the corresponding transfer integral deviates from the transfer integral in the crystal grain (roughly 80.000 pairs for 20.000 molecules per realization). Third, the correlation of the average energy of two molecules forming a grain-boundary pair and the corresponding angle difference between the *a*-directions of the grains is displayed in a two-dimensional histogram, see **Figure S16**. The color scale is correctly normalized to the probability for the difference of two uniformly distributed angles between 0° and 360°, which linearly decreases from 0° to 360°. While for the activation barriers ($E > 0$) no clear correlation is observed, repeating the procedure only for the valleys we find that no energy valleys are created for relative angles up to 60° and between 300° and 360°, while for relative angles around 180° it is very likely that deep valleys at the grain boundary are generated. We assign those deep trap states to the asymmetry induced by the molecular torsion and the resulting dipole moment.

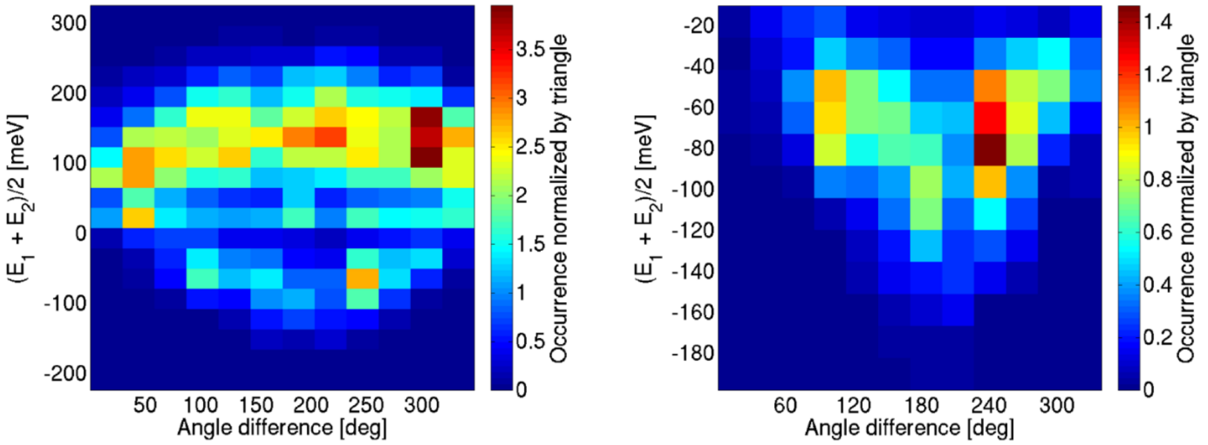

Figure S16: Correlation between the mean energy for molecular pairs across grain boundaries and the angle difference of the respective *a*-directions of the two crystal grains. The two-dimensional histograms have been normalized to the probability for the difference between two uniformly distributed angles in the range between 0° to 360°. While for the energy barriers (E > 0) no clear correlation is shown (left) concentrating only on the traps (E < 0) we often find deep traps at grain-angles around 180°.

2.8. Energy landscape in the HOMO

With respect to the energy landscape we have also checked for one 10x10 morphology that the landscape is inverted when we consider partial charges for the cationic molecule in **Equation 2**. As a result, energetic barriers turn into valleys and vice versa, as shown in **Figure S17**. This is to be expected when the charged molecule is well described by a point charge interacting with surrounding multipole moments. We note however, that in our case the DOS is not symmetric around E = 0, e.g. for the anionic system we only see a clear exponential decay for the energy valleys but not for the barriers, so that the energy valleys in the cationic system will not display an exponential decay.

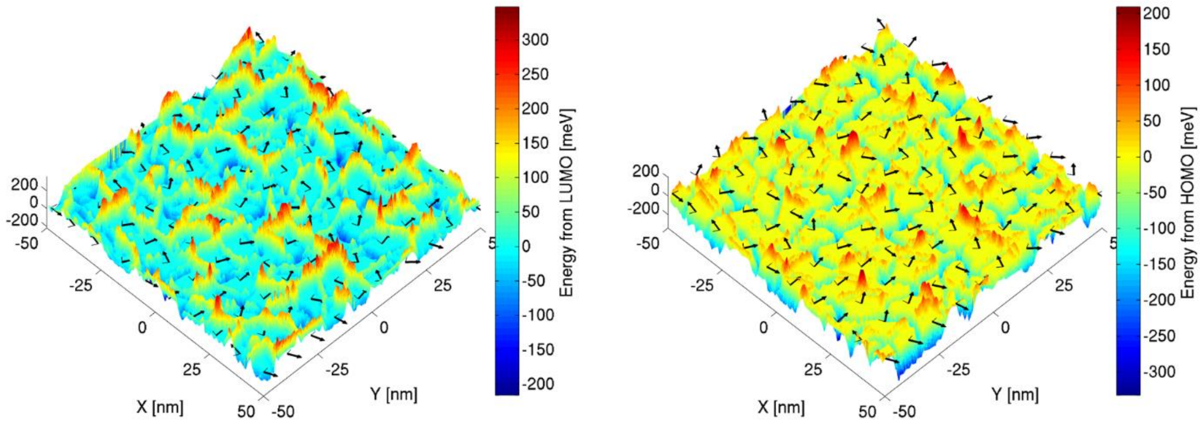

Figure S17: Electrostatic energy landscape for one electron, i.e. the electron affinity, EA, (left) and electrostatic energy landscape for one hole, i.e. the ionization potential, (right) in a monolayer of 10x10 crystal grains per $(100nm)^2$.

3. Theoretical description of charge transport

We have also evaluated transport of one electron through the derived energy landscape in an applied electric field as detailed below.

3.1. Calculation of transfer integrals

Besides the electrostatic energy landscape discussed in the last section, the electronic coupling between spatially close molecules, which is quantified by transfer integrals, is mainly responsible for the charge transport characteristics across grain boundaries. Due to the large number of molecular pairs (approximately 80.000 per studied morphology if only those with an internal center-of-mass distance of less than 2 nm are considered), for which transfer integrals need to be calculated, we applied the semi-empirical ZINDO (Zerner's Intermediate Neglect of Differential Overlap) method[18,19] instead of the commonly used but computationally more expensive DFT.

As mentioned before, in case of the proposed crystal structure (θ = 30°, ϕ = 190°, Ψ = 0°), the ratio of the largest transfer integral (in *a*-direction) to the second largest transfer integral (in *a*+*b*-direction) amounts to $J_a/J_{a+b} = 1.4$ leading to an effective two-dimensional charge transport within the crystal grains with absolute values of J_a = 118.33 meV (internal center-of-mass distance $d = |\vec{a}|$ = 0.546 nm) and J_{a+b} = 85.75 meV ($d = |\vec{a} + \vec{b}|$ = 0.817 nm). The electronic couplings along the *a*-*b*- and the *b*-directions are significantly smaller with $J_{a\text{-}b}$ = 0.45 meV ($d = |\vec{a} - \vec{b}|$ = 1.099 nm) and J_b = 0.02 meV ($d = |\vec{b}|$ = 0.854 nm). We note, that very similar transfer integrals are obtained using DFT (BP86/def2-TZVP) instead of ZINDO (J_a = 119.25 meV, J_{a+b} = 83.91 meV, $J_{a\text{-}b}$ = 0.71 meV and J_b = 0.10 meV).

As can be seen in **Figure S18**, the presence of grain boundaries leads to additional transfer integrals, which are smaller than the two largest transfer integrals within the crystal grains, J_a and J_{a+b}, in virtually all cases. The distance dependency of those additional transfer integrals can be modelled by an exponential function ($\sim e^{-d/\Lambda}$) with a characteristic wave function decay length of about Λ = 0.2 nm. We note again, that assuming the same crystal structure as for PDI-FCN$_2$ (θ = 30°, ϕ = 260°, Ψ = 100°), the ratio of the largest to the second largest transfer integral amounts to J_a/J_{a+b} = 366 leading to an effective one-dimensional charge transport within the crystal grains with J_a = 58.61 meV, J_{a+b} = 0.16 meV, $J_{a\text{-}b}$ < 0.01 meV and J_b < 0.01 meV.

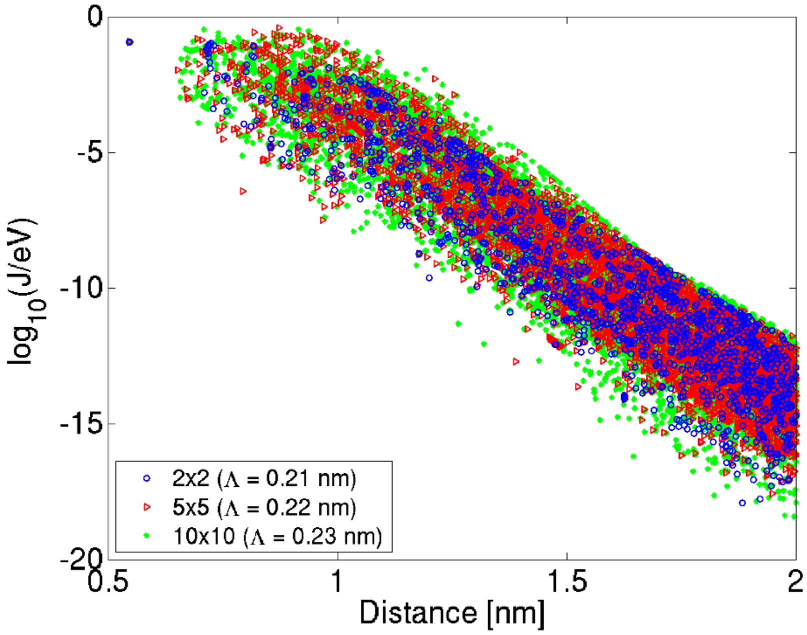

Figure S18: Transfer integrals for all molecular pairs with an internal center-of-mass distance (*d*) of less than 2 nm within a monolayer of *N*x*N* crystal grains (*N* = 2, 5, 10) per $(100\text{nm})^2$ using the semi-empirical ZINDO method. To improve statistics, two realizations of grain formation (random *a*-directions) are taken into account for each *N*x*N*. An exponential decay ($\sim e^{-d/\Lambda}$) of the transfer integrals is observed with Λ being the wave function decay length.

3.2. Charge Transport Simulations

3.2.1. Charge carrier trajectory

In **Figure S8** (same as Figure 3 in the main text) and **Movie S2** a typical electron trajectory is visualized for the 5x5 morphology with a mobility of μ = 1.06 cm^2/Vs. The white mesh characterizes all molecules that are occupied by the electron at least once during the total simulation time of 70 ps. The electron is injected in the area labelled “start”. It mainly travels against the applied electric field (due to its negative charge) and has to circumvent activation barriers (red), see (a) and (c), is partially trapped in the valleys (dark blue), see (b) and (d), and finally trapped between three barriers, see (e) or “end”. On the upper right-hand side exemplary energy profiles for a barrier (along the red dashed arrow, see (a)) and a valley (along the blue dashed arrow, see (d)) are shown. It is noteworthy that the electron spends about 21 ps (30 % of the total time) in the deep trap (d).

3.2.2. Charge carrier mobilities

Having discussed all ingredients to the Levich-Jortner hopping rates (**Equation 1**), in the following we focus on the results of our charge transport simulations to determine average mobilities. As already mentioned above, mobilities need to be calculated for different directions of the applied electric field $\vec{F}$ (field strength $F = |\vec{F}| = 1\mathrm{V}/100\mathrm{nm}$), since the sample can be oriented randomly in the OFET channel. Thus, we considered all possible field directions within the two-dimensional transport plane (from 0° to 180° with respect to the *x*-axis in steps of 10°). For each of those 18 field directions 50 Monte-Carlo simulations for a single, randomly injected electron were performed applying periodic boundary conditions in the transport plane and averaged to yield the directional mobility $\mu = (\vec{v} \cdot \vec{F})/F^2$ with $\vec{v}$ being the mean velocity of the electron. Finally, averaging over all 18 directional mobilities as well as over two realizations of grain formation (random *a*-directions to improve statistics) a mean mobility for each *N*x*N* morphology can be obtained together with a corresponding error bar from the standard deviation, see **Figure S19**. The simulation time was adapted in such a way that the electron reaches mean travelling distances of around 100 nm (= box length). All calculations were done with VOTCA.[17]

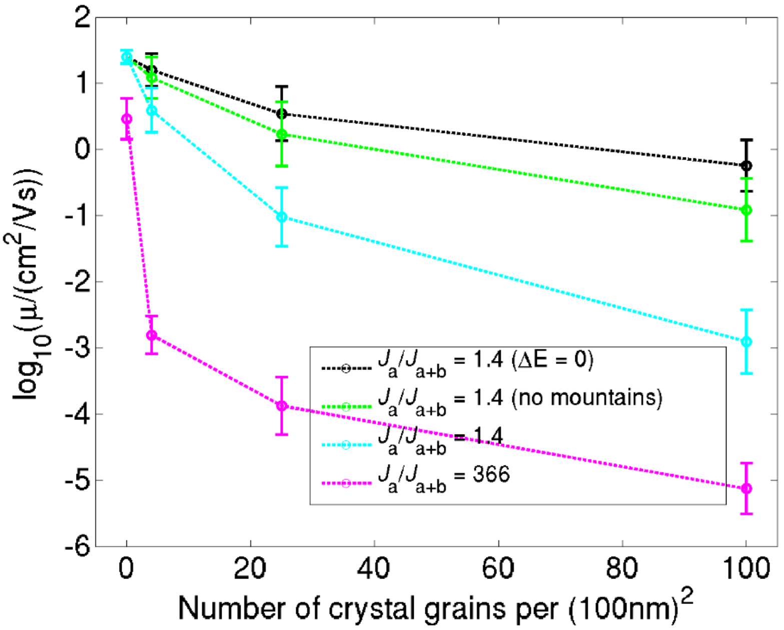

Figure S19: Mobilities within a monolayer of *N*x*N* crystal grains (*N* = 2, 5, 10) per (100nm)^2 as well as within a perfect crystal for one electron at temperature T = 300 K from Levich-Jortner hopping rates for two-dimensional charge transport within the crystal grains (transfer integral ratio J_a/J_{a+b} = 1.4; black, green, and cyan data points) as well as for one-dimensional charge transport (J_a/J_{a+b} = 366; magenta). Turning off the energetic disorder (black) or neglecting energetic barriers (green) in the case of two-dimensional transport increases mobilities by several orders of magnitude. Mean values and error bars are calculated from averaging over 18 external electric field directions within the transport plane from 0° to 180° in steps of 10° with respect to the *x*-axis and 50 randomly chosen electron injection points for each field direction. The field strength is *F* = 1V/100nm.

In **Figure S19** mean mobilities and corresponding error bars are shown for all investigated *N*x*N* grain-boundary morphologies (*N* = 2, 5, 10) as well as for a perfectly crystalline morphology. We note that the absolute values of the calculated mobilities are expected to be too large since dynamic (thermal) disorder was not taken into account in our calculations, which in general would decrease all mobilities. Furthermore we have not considered charge-carrier density effects in our simulations which however is expected to not play a major role as discussed in the main text and below. Nevertheless we focus on the discussion of trends in the following.

As already described above, assuming two-dimensional charge transport in a perfectly crystalline morphology (J_a/J_{a+b} = 1.4, cyan data points), a simulated mean mobility of 25.13 cm^2/Vs with a standard deviation of 5.86 cm^2/Vs (23 %) is obtained. The corresponding mobility tensor is shown in **Figure S20** (left). This calculated standard deviation is in good agreement with the standard deviation of the measured mobility in the highly crystalline DMP:toluene (1:3) sample of about 17 %. As expected, the mean mobility decreases significantly with increasing number of crystal grains per area: by one order or magnitude for the 2x2 morphology (μ(2x2) = 3.94 cm^2/Vs), by more than two orders of magnitude for the 5x5 morphology (μ(2x2) = 0.10 cm^2/Vs) and by four orders of magnitude for the 10x10 morphology (μ(10x10) = 0.001 cm^2/Vs) relative to the mobility for a perfectly crystalline morphology. It is noted, that corresponding mobilities using the Marcus hopping model are smaller throughout (e.g. 4.05 cm^2/Vs for the perfectly crystalline morphology in case of J_a/J_{a+b} = 1.4), since the corresponding rates are roughly proportional to $e^{-\lambda^{\text{int}}/\,k_BT}$ with $\lambda^{\text{int}} = 0.23\text{ eV} \gg \lambda^{\text{out}} = 0.05\text{ eV}$ and thus smaller than the Levich-Jortner rates, see **Equation 1**.

In order to investigate the influence of energetic barriers and valleys on the mobilities, we first neglected all barriers by setting all energies with $E > 0$ to $E = 0$ in the simulated DOS, i.e. only valleys are taken into account (no barriers, J_a/J_{a+b} = 1.4, green data points). Then, we additionally neglected all valleys, i.e. we turned off the energetic disorder completely ($\Delta E = 0$, J_a/J_{a+b} = 1.4, black data points). With respect to the full disorder, neglect of energetic barriers increases mobilities by about one to two orders of magnitude for all *N*x*N* grain-boundary morphologies (the larger *N*x*N* the larger the increase). Additional neglect of energetic valleys increases those mobilities further, however, by less than one order of magnitude. Therefore we conclude, that energetic barriers have a larger (limiting) influence on mobilities (by blocking pathways in the monolayer) than energetic valleys or trap states. As discussed in the main text the impact of a high charge carrier density would similarly lead to a smearing out of the energy valleys. We also note, that in the case of no energetic disorder the small decrease in mobilities for increasing *N*x*N* is only due the introduction of an increasing number of small transfer integrals at the grain boundaries. This shows that for lower degree of crystallinity, the increased

energetic disorder (especially the barriers) has a stronger limiting effect on the charge transport than the larger amount of weak transfer integrals across grain boundaries.

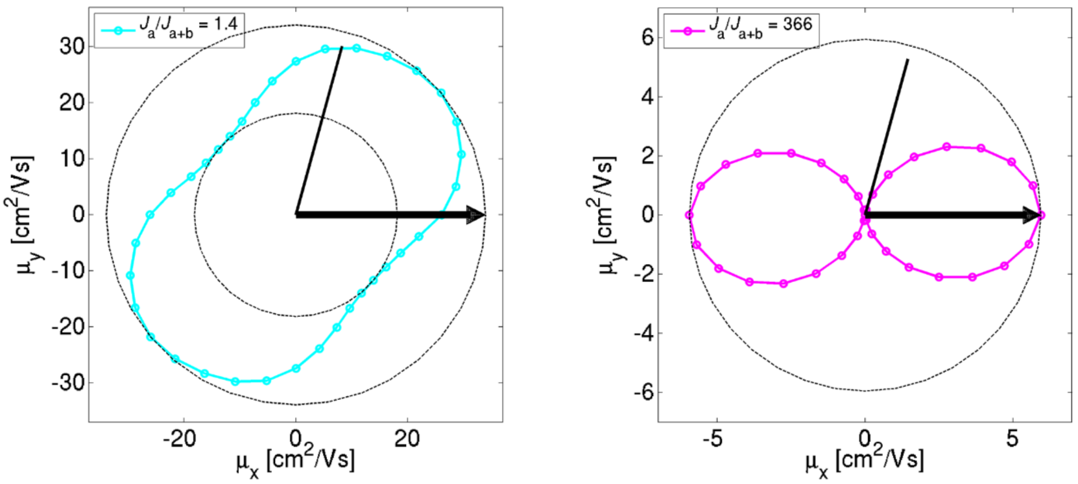

Figure S20: Comparison of mobility tensors within a perfect crystal for one electron at temperature T = 300 K from Levich-Jortner hopping rates for two-dimensional charge transport (J_a/J_{a+b} = 1.4; left) as well as for one-dimensional charge transport (J_a/J_{a+b} = 366; right). 18 external electric field directions within the transport plane 0° to 180° in steps of 10° with respect to the *x*-axis (= *a*-direction) were considered. The field strength is *F* = 1V/100nm. The black arrow along the *x*-axis indicates the *a*-direction, whereas the black line indicates the *a*+*b*-direction.

Last we compare the two-dimensional charge transport (J_a/J_{a+b} = 1.4, cyan data points) discussed above to the one-dimensional charge transport within the crystal grains (J_a/J_{a+b} = 366, magenta data points). As already described, for a perfectly crystalline morphology exhibiting one-dimensional charge transport we calculated a mobility of 2.92 cm^2/Vs with a large standard deviation of 2.08 cm^2/Vs (71 %), that is due to the strong dependence of the mobility on the direction of the applied electric field in case of one-dimensional charge transport, see **Figure S20** (right). Since this large standard deviation is not in agreement with the corresponding measured value of 17 % for the highly crystalline DMP:toluene (1:3) sample we proposed a two-dimensional charge transport morphology, see above. For all investigated *N*x*N* grain-boundary morphologies (*N* = 2, 5, 10) mobilities based on one-dimensional charge transport are smaller than the corresponding mobilities assuming a two-dimensional charge transport by about three orders of magnitude. We have checked that this is not mainly due to the energetic disorder, which in fact is rather similar for the one- and two-dimensional charge transport, e.g. the standard deviations (STDEV) of the DOS in the (2x2, 5x5, 10x10) morphologies are (31, 47, 64) meV for the one- and (27, 43, 61) meV for the two-dimensional charge transport with very similar shapes of the DOS. The reason for larger mobility in case of two transport directions is due to the possibility to sample the whole grain boundary and choose the best grain boundary pair with respect to the transfer integral for crossing, whereas in case of one-dimensional charge transport the charge carrier has to cross the grain boundary at one fixed (probably unfavorable) grain-boundary pair since it is not allowed to move perpendicular to it. This is also in line with the observation that the mobility for one-dimensional charge

transport decreases drastically when going from a perfectly crystalline morphology (μ(crystal) = 2.92 cm^2/Vs) to a 2x2 morphology (μ(2x2) = 0.002 cm^2/Vs). We note that including dynamic disorder would allow the bad transfer integrals across a grain boundary to increase on the time scale of molecular fluctuations due to improved orbital overlap which will decrease the difference between one- and two-dimensional charge transport. However, the corresponding energy landscape will not change, as this would require major reorientation of several molecules, such that the trends related to the energetics discussed above should remain.

As we wanted to employ *ab-initio* simulations at atomistic resolution, the regime for the degree of crystallinity we could cover considering roughly 20.000 molecules in our 100 nm x100 nm substrate ranges from 4 x 10^8 to 2.25 x 10^9 crystal grains per mm^2. Although this is a significantly lower degree of crystallinity compared to what is found by optical methods in the experiment, we believe that the trends from the simulation reveal interesting aspects about the transport mechanism. However, it is clear that the simulated mobilities we obtained by allowing the additional electron to sample a channel of length L = 100 nm will reduce towards longer channel lengths up to L = 50 µm used in experiment. A simple way to capture the effect of different channel lengths can be achieved via **Equation 3**, where the mobilities μ_k are taken from different realizations of a morphology with given crystallinity, for different injection points and different electrical field directions.

$$\mu_M = \frac{1}{\sum_{k=1}^{M} \mu_k^{-1}} \qquad \textbf{Eq.3}$$

Although all mobilities from the same morphology realization are not completely independent, this procedure is roughly equivalent to connecting M independent simulation boxes in series allowing to scale up the channel length to 100 nm x M. We have done this for one 2x2 grain boundary morphology with M = 1 to 900, which is equivalent to channels of length L = 100 nm to 90 µm, randomly drawing μ_k from the distribution of simulated mobilities with a trajectory length of roughly 100 nm including 50 injection points and 18 different electrical field directions. This reveals that for the highest degree of crystallinity simulated, corresponding to 4x10^8 grains per mm^2 an experimental mobility around 10^{-3} cm^2/Vs would be expected, see **Figure S21**. The experimentally found mobilities are several orders of magnitude larger because the degree of crystallinity is much larger (up to 2 grains per mm^2) compared to what was reachable in our *ab-initio* simulations.

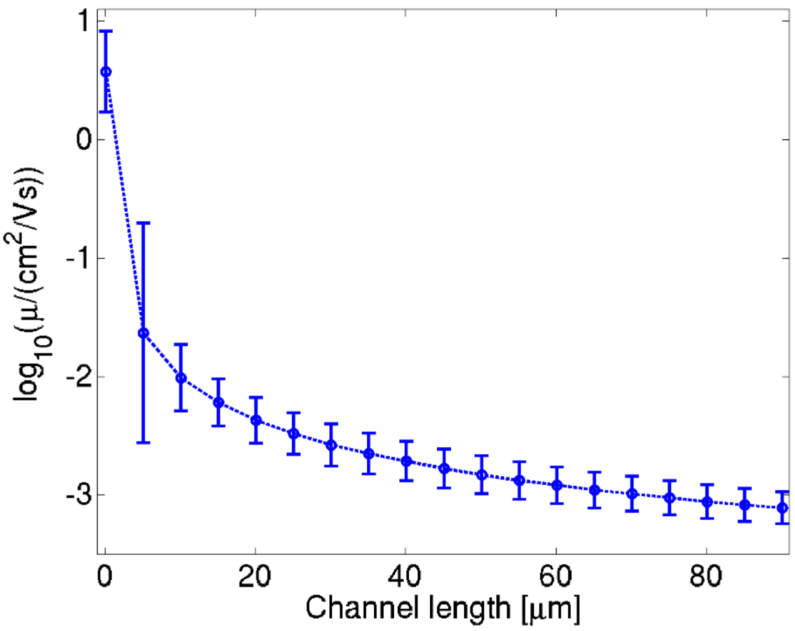

Figure S21: Mobilities for 2x2 crystal grains per $(100nm)^2$ are scaled up via **Equation 3** from the distribution of simulated mobilities with 100 nm trajectory length for 50 injection points and 18 electrical field directions. Correspondingly, for 4 x 10^8 grains per mm^2 and the experimental channel length of L = 50 µm one would expect mobilities of 10^{-3} cm^2/Vs due to low degree of crystallinity. Much larger mobilities have been achieved in experiment due to higher degree of crystallinity.

References

1 Hulea, I. N. *et al.* Tunable Frohlich polarons in organic single-crystal transistors. *Nature Materials* **5**, 982-986, doi:10.1038/nmat1774 (2006).

2 Kalb, W. L., Haas, S., Krellner, C., Mathis, T. & Batlogg, B. Trap density of states in small-molecule organic semiconductors: A quantitative comparison of thin-film transistors with single crystals. *Physical Review B* **81**, 155315, doi:10.1103/PhysRevB.81.155315 (2010).

3 Kalb, W. L. & Batlogg, B. Calculating the trap density of states in organic field-effect transistors from experiment: A comparison of different methods. *Physical Review B* **81**, 035327, doi:10.1103/PhysRevB.81.035327 (2010).

4 Hansen, N. H. *et al.* Anisotropic electron mobility studies on Cl2-NDI single crystals and the role of static and dynamic lattice deformations upon temperature variation. *arXiv:1501.01856v3* (2016).

5 *TURBOMOLE V7.0 2015, a development of University of Karlsruhe and Forschungszentrum Karlsruhe GmbH, 1989–2007, TURBOMOLE GmbH since 2007; available from http://www.turbomole.com.*

6 Becke, A. D. DENSITY-FUNCTIONAL THERMOCHEMISTRY .3. THE ROLE OF EXACT EXCHANGE. *Journal of Chemical Physics* **98**, 5648-5652, doi:10.1063/1.464913 (1993).

7 Weigend, F. & Ahlrichs, R. Balanced basis sets of split valence, triple zeta valence and quadruple zeta valence quality for H to Rn: Design and assessment of accuracy. *Physical Chemistry Chemical Physics* **7**, 3297-3305, doi:10.1039/b508541a (2005).

8 Becke, A. D. DENSITY-FUNCTIONAL EXCHANGE-ENERGY APPROXIMATION WITH CORRECT ASYMPTOTIC-BEHAVIOR. *Physical Review A* **38**, 3098-3100, doi:10.1103/PhysRevA.38.3098 (1988).

9 Perdew, J. P. DENSITY-FUNCTIONAL APPROXIMATION FOR THE CORRELATION-ENERGY OF THE INHOMOGENEOUS ELECTRON-GAS. *Physical Review B* **33**, 8822-8824, doi:10.1103/PhysRevB.33.8822 (1986).

10 Klamt, A. & Schuurmann, G. COSMO - A NEW APPROACH TO DIELECTRIC SCREENING IN SOLVENTS WITH EXPLICIT EXPRESSIONS FOR THE SCREENING ENERGY AND ITS GRADIENT. *J. Chem. Soc.-Perkin Trans. 2*, 799-805, doi:10.1039/p29930000799 (1993).

11 Jones, B. A. *et al.* High-mobility air-stable n-type semiconductors with processing versatility: Dicyanoperylene-3,4 : 9,10-bis(dicarboximides). *Angewandte Chemie-International Edition* **43**, 6363-6366, doi:10.1002/anie.200461324 (2004).

12 Grimme, S., Antony, J., Ehrlich, S. & Krieg, H. A consistent and accurate ab initio parametrization of density functional dispersion correction (DFT-D) for the 94 elements H-Pu. *Journal of Chemical Physics* **132**, 154104, doi:10.1063/1.3382344 (2010).

13 Fratini, S., Mayou, D. & Ciuchi, S. The Transient Localization Scenario for Charge Transport in Crystalline Organic Materials. *Advanced Functional Materials* **26**, 2292-2315, doi:10.1002/adfm.201502386 (2016).

14 Bredas, J. L., Beljonne, D., Coropceanu, V. & Cornil, J. Charge-transfer and energy-transfer processes in pi-conjugated oligomers and polymers: A molecular picture. *Chemical Reviews* **104**, 4971-5003, doi:10.1021/cr040084k (2004).

15 Jortner, J. TEMPERATURE-DEPENDENT ACTIVATION-ENERGY FOR ELECTRON-TRANSFER BETWEEN BIOLOGICAL MOLECULES. *Journal of Chemical Physics* **64**, 4860-4867, doi:10.1063/1.432142 (1976).

16 Marcus, R. A. ELECTRON-TRANSFER REACTIONS IN CHEMISTRY - THEORY AND EXPERIMENT. *Reviews of Modern Physics* **65**, 599-610, doi:10.1103/RevModPhys.65.599 (1993).

17 Ruhle, V. *et al.* Microscopic Simulations of Charge Transport in Disordered Organic Semiconductors. *Journal of Chemical Theory and Computation* **7**, 3335-3345, doi:10.1021/ct200388s (2011).

18 Ridley, J. & Zerner, M. INTERMEDIATE NEGLECT OF DIFFERENTIAL OVERLAP TECHNIQUE FOR SPECTROSCOPY - PYRROLE AND AZINES. *Theoretica Chimica Acta* **32**, 111-134, doi:10.1007/bf00528484 (1973).
19 Kirkpatrick, J. An approximate method for calculating transfer integrals based on the ZINDO Hamiltonian. *International Journal of Quantum Chemistry* **108**, 51-56, doi:10.1002/qua.21378 (2008).
