# Supplementary figures and images for: Energy barriers at grain boundaries dominate charge carrier transport in an electron-conductive organic semiconductor
Source: Sci Rep. 2018 Oct 5;8:14868. doi: 10.1038/s41598-018-33308-y (PMC6173704; doi:10.1038/s41598-018-33308-y)

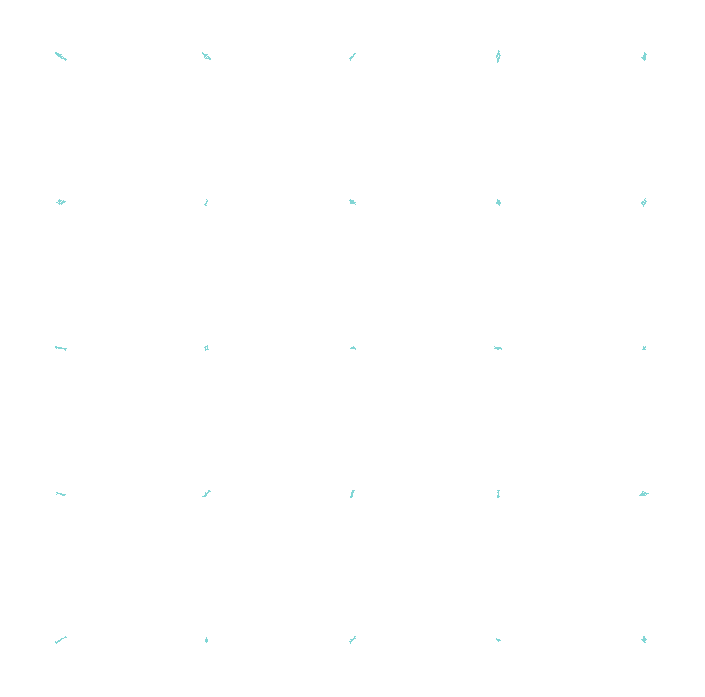

Supplement: Supplementary file 3 — Supplementary Movie 1 [file 41598_2018_33308_MOESM3_ESM.gif]
